# Active robotic assistance for standing and sitting: experimental evaluation of handle trajectories

**DOI:** 10.1186/s12984-025-01849-9

**Published:** 2026-02-05

**Authors:** Marko Ackermann, Lizeth H. Sloot, Katja Mombaur

**Affiliations:** 1 Institute for Anthropomatics and Robotics, Karlsruhe Institute of Technology (KIT), Adenauerring 12, 76131 Karlsruhe, BW Germany; 2 Translational and Clinical Research Institute, Newcastle University, Newcastle upon Tyne, Tyne and Wear, NE1 7RU UK; 3 Systems Design Engineering Department, University of Waterloo, 200 University Avenue West, Waterloo, ON N2L 3G1 Canada

## Abstract

**Background:**

Standing up and sitting down are important activities of daily living, but require large leg moments that often exceed the muscle strength of older adults. Some robotic rollators are designed to provide standing-up and sitting-down assistance through actuated handles or armrests to reduce the loads on the legs, but it is still unclear how they should move. There is limited information on appropriate assistance trajectories and their effects on the body during standing up and sitting down.

**Methods:**

We designed four physiological, scalable and parameterized handle trajectories based on unassisted shoulder movement that can be readily implemented in robotic assistive devices, and evaluated their effect on leg loading, energy input, handle forces and perceived assistance in 15 healthy younger adults. We created a robotic assistance simulator device equipped with moving handles to compare the trajectories to static handles (representing a conventional rollator), and collected full-body motion, ground reaction forces, handle forces and scored perceived assistance.

**Results:**

The proposed handle trajectories substantially decreased leg loads compared to the static handle assistance (non-moving handle), with the two best-performing trajectories reducing the peak hip extension moment by over 70% and the peak knee extension moment by over 50% during standing up and sitting down. This is associated with an increase in peak vertical handle forces of over 30%, with the total bilateral vertical forces reaching up to 60% of body weight, and a decrease in peak horizontal force of more than 50%. The subjective participants’ perception reflected the lower limb mechanical load. The handle velocity was shown to play a secondary role within the investigated range.

**Conclusion:**

The proposed support trajectories can be scaled to the person’s anthropometry and readily implemented in robotic assistive devices, and were shown to substantially reduce leg loading, potentially improving life quality of individuals with difficulties in standing up. However, the large vertical handle forces and thus upper body demand during moving-handle assistance is a trade-off with relieving the lower limb load. This work provides a comprehensive foundation for the design of the necessary further experimental assessments with the target population.

## Background

Standing up and sitting down (STSs) are important activities of daily living (ADLs), being performed more than 50 times per day [1], and guarantee an independent life-style by enabling other activities such as walking. However, they are among the most demanding tasks in terms of lower extremity loads [2]. Standing up requires larger peak knee and hip extension moments than other daily living activities such as walking or climbing stairs [3, 4], while sitting down requires only slightly smaller peak hip and knee extension moments [5].

Difficulties in standing up and sitting down substantially affect the quality of life, particularly in older adults as their muscle capacity deteriorates with ageing [6]. Gross et al. [7] show a reduction of 35% and 55% in maximal hip and knee extension moments in older adults, while Hortobagyi and colleagues [8] conclude that healthy older adults approximate their maximal strength capacity while rising from a chair. This shows how challenging standing up can be without compensatory movement strategies or external assistance. One study showed that about 48% of a group of healthy older adults were unable to stand up without the help of the hands when the seat height was set at about the knee height, with 8% using the arms to generate additional momentum, 18% pushing the thighs or chair seat with the hands, while 22% was not able to stand independently at all [9]. Difficulties in standing up are not limited to older adults. Davidson and colleagues [10] report that more than 80% of people with osteoarthritis were unable to stand up without the help of armrests.

Passive rollators are prescribed to provide support and improve postural stability during walking in patients with neuromuscular disorders, muscular weakness and balance impairments [11]. In practice, they are also frequently used to support standing up and sitting down [12], particularly if other assistance such as hand rails and armrests are not available [13, 14]. However, as the body is behind the rollator handles, it is difficult to transfer vertical forces from the handles to the trunk to support lifting the body up. Furthermore, as these rollators are often light and their bases of support are reduced and located in front of the body, the maximal horizontal forces that can be applied at the handles without tipping the rollator over are limited. Thus, while rollators are used to support standing up and sitting down [14], the use of walking aids and assistive devices, including rollators, is suggested to be a risk factor for falling [15, 16], and is associated with a high risk of severe injuries in older adults [17, 18].

Robotic rollators, or smart walkers, use active robotic systems to extend the assistive capabilities of passive rollators, providing additional functionalities such as navigation, maneuverability improvement, fall prevention, gravity compensation on slopes, obstacle avoidance, health monitoring and partial weight support, as well as STS assistance [19, 20]. Seven of the rollators surveyed in [20] include active STS assistance systems: *MOBIL* [21, 22], *MONIMAD* [23, 24], *Chugo’s group walker* [25–27], *WAR* [28], *SMW* [29, 30], *Standing Assistive Walker* [31, 32], and *MOBOT* [33–35]. The assistance is provided by handles (*MONIMAD*, *WAR*, *MOBOT*) or forearm support/armrests (*MOBIL*, *Standing Assistive Walker*, *SMW*) moving in the sagittal plane with mostly two actuated degrees of freedom and bilateral symmetry with respect to the sagittal plane. As described in [20], the control approaches include motion control, force control, as well as switching strategies based on the estimated postural state, lower limb loads or stability criteria. Motion control approaches range from positioning the rollator in front of the user and activating the brakes [36] to implementing moving handles with guiding trajectories. In [29] and [30], for instance, the authors propose and briefly compare two predefined trajectories, one with no inclination of the 3-DoF forearm support and the other inclining it forward during the sit-to-stand assistance motion. Unfortunately, no specific information on the trajectories of the forearm support is provided. Pasqui and colleagues [23, 24] define s-shaped, smooth trajectories that minimize jerk while approximating recorded assisted STS transfers by cubic splines. Kawazoe et al. [31] used trajectories based on prior data collected with an experienced healthcare professional in their motion control strategy. Geravand and colleagues [35], in turn, propose reference trajectories for *MOBOT* obtained by solving an optimal control problem. Even the implementation of more complex switching control strategies often require reference or nominal trajectories for their controllers such as in [32].

Unfortunately, few details are reported of these trajectories, making them difficult to reproduce. No studies have compared different trajectories directly. Moreover, the effect of robot-user interaction on posture, loading and balance have hardly been evaluated. In fact, in their review paper [20], Geravand and colleagues emphasize the “*lack of formal evaluation studies with patients investigating the benefits of developed systems and functionalities from a clinical and user perspective*”, and indicate that acceptance studies are missing in the literature. Additionally, there is very limited information on how those trajectories would be scaled or adapted to different individuals.

Considering this gap in the literature, the aim of this study is threefold: (i) propose simple, parameterized, and scalable trajectories based on unassisted sit-to-stand and stand-to-sit patterns and simple geometric shapes that could be readily used in other studies and implemented in STS assistance devices; (ii) provide a baseline comparison of these assistance trajectories in healthy younger adults in terms of leg loading, handle forces and subjective perceived support; (iii) evaluate the effect of handle speed on these outcome domains. We hypothesize that: (i) providing moving-handle assistance through the proposed trajectories will reduce the lower limb loading during standing up and sitting down compared to the support provided by static handles in conventional rollators; (ii) handle trajectories closer to the reference shoulder trajectories for unassited STS, which have a curved shape, will lead to better performance compared to the straighter trajectory shapes tested; (iii) handle speed will affect substantially the user’s biomechanical response.

## Methods

We performed experiments with a robotic assistance device to evaluate four assistance trajectories (proposed on the basis of previously measured shoulder trajectories during unassisted standing up and sitting down) and compare them to a static handle. The collections were performed in the HCMR motion capture lab at Heidelberg University with 15 young, healthy participants. Kinematics, ground reaction forces, and handle forces were collected, and the subjective perception on assistance and safety was assessed using a questionnaire. “Robotic assistance simulator device” section introduces the Robotic Assistance Simulator device used to reproduce the proposed trajectories. “Proposed assistance trajectories” section describes the proposed handle trajectories. “Experimental set-up” section details the experimental setup. “Experimental protocol” section describes the protocol, and data collection. Finally, “Data evaluation” section describes the data treatment, outcome metrics, and statistical analysis.

### Robotic assistance simulator device

**Fig. 1:**
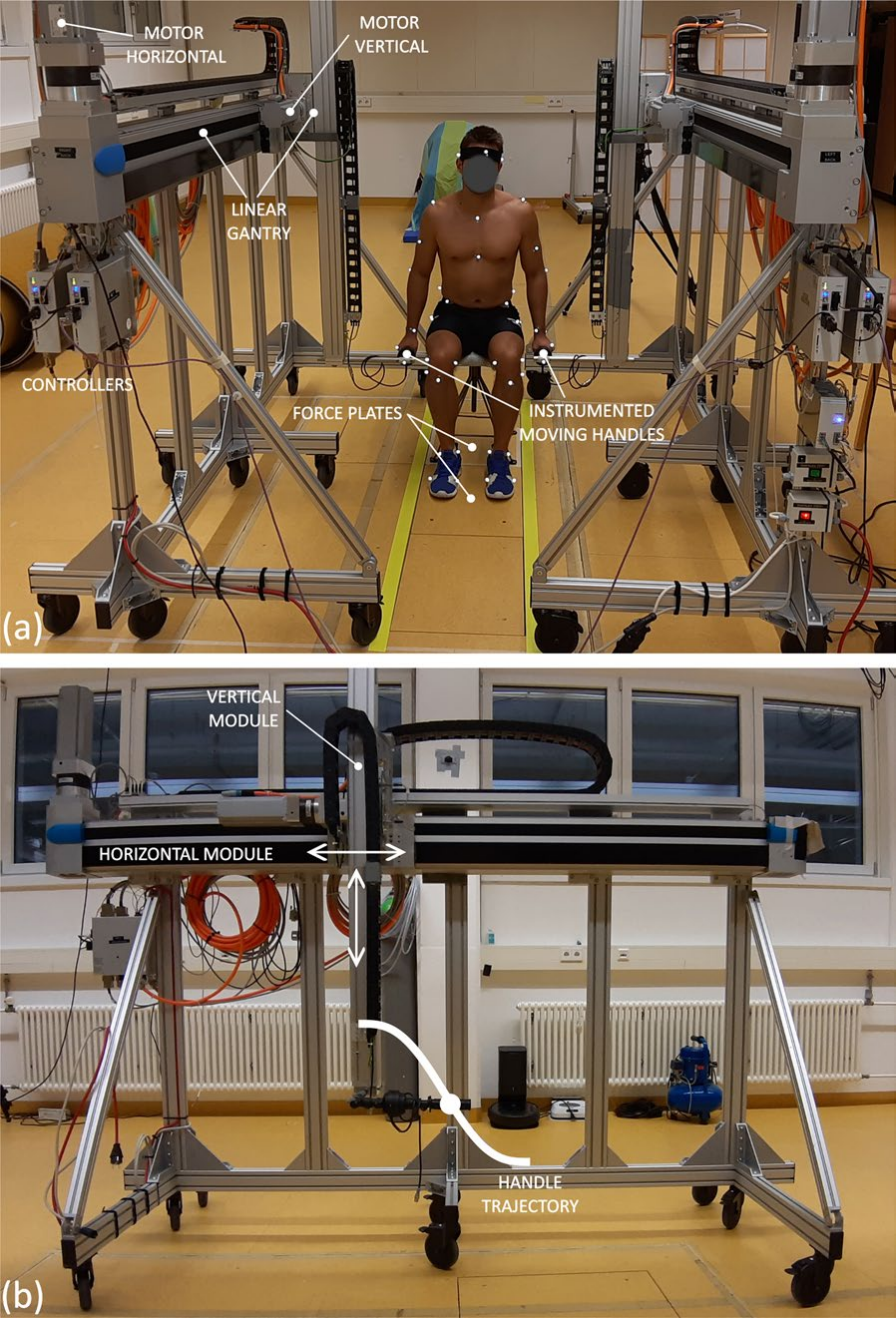
Robotic Assistance Simulator (RAS) device and experimental setup (**a**, *top*) in the motion capture lab at Heidelberg University. Handles are aligned with the seated wrist height at the start of the movement, with the feet on one force plate and the stool (set to approximately knee height) on the other force plate. 49 markers are placed to track the motion. The RAS device can apply arbitrary trajectories to the instrumented handle in the sagittal plane (**b**, *bottom*)

The Robotic Assistance Simulator (*RAS*) device (see Fig. 1), designed and constructed within the Heiage project at Heidelberg University, allows to experimentally investigate the effect of different assistance strategies for actuated handles. Among other types of controllers, it can move bilateral handles along prescribed trajectories (Fig. 1b) contained in the sagittal plane, emulating the support provided by robotic rollators with moving handles. Each handle is instrumented with a 6-axis load cell (FT300, Robotiq, Lévis, QC, Canada; sampled at 100 Hz).

The device encompasses two Cartesian robots, placed on each side of the participant, each containing a 2D linear gantry assembly (2D Linienportal, YXCL-4, Festo SE & Co. KG, Esslingen, Germany) composed of vertical and horizontal modules, with maximal horizontal and vertical loads of 2500 N and 1000 N, respectively, attached to a wheeled aluminum frame. The horizontal module, with a max. working stroke of 2000 mm, comprises a linear toothed belt axis (EHMY-LP-EGC-185-TB-KF-2000-L) powered by a servomotor (EMME-AS-100-M-HS-AMB, 7.5 Nm, 3000 rpm, 2000 W) connected to a 3:1 gearbox (EMGA-120-P-G3-SAS-100). The vertical module, with a max. working stroke of 800 mm, comprises a linear spindle axis (EHMZ-DGEA-40-TB-KF-800-L) powered by a servomotor (EMME-AS-80-M-LS-AMB, 3.5 Nm, 3000 rpm) connected to a 3:1 gearbox (EMGA-80-P-G3-EAS-80). The four motors are equipped with brakes and are controlled by digital controllers (CMMP-AS-C5-3A-M0, control loop frequency 8 kHz) through a Speedgoat real-time target machine (Baseline, Speedgoat GmbH, Liebefeld, Switzerland) via the CANopen communication protocol. Matlab/Simulink (The Mathworks, Inc., Natick, MA, USA) is used to generate the handles’ position and velocity profiles over time by means of the Speedgoat real-time machine. In this study, the *RAS* device was used to generate the prescribed bilateral velocity profiles corresponding to each of the proposed assistance trajectories introduced in “Proposed assistance trajectories” section.

### Proposed assistance trajectories

An appropriate handle motion follows the shoulder joint, as extended, vertical arms provide an effective and low-energy means to transfer vertical assistive forces from handles to the trunk. For this reason, we explored different handle trajectory shapes and velocity profiles inspired by shoulder motion during unassisted standing and sitting. We first determined a reference velocity profile based on shoulder motion using a reference dataset in unassisted sit-to-stand and stand-to-sit motions. Based on these reference velocity profiles, we created the four assistance trajectories, each generated in the form of scalable, parameterized horizontal and vertical velocity profiles. These profiles are described below.

#### Reference trajectories

**Fig. 2:**
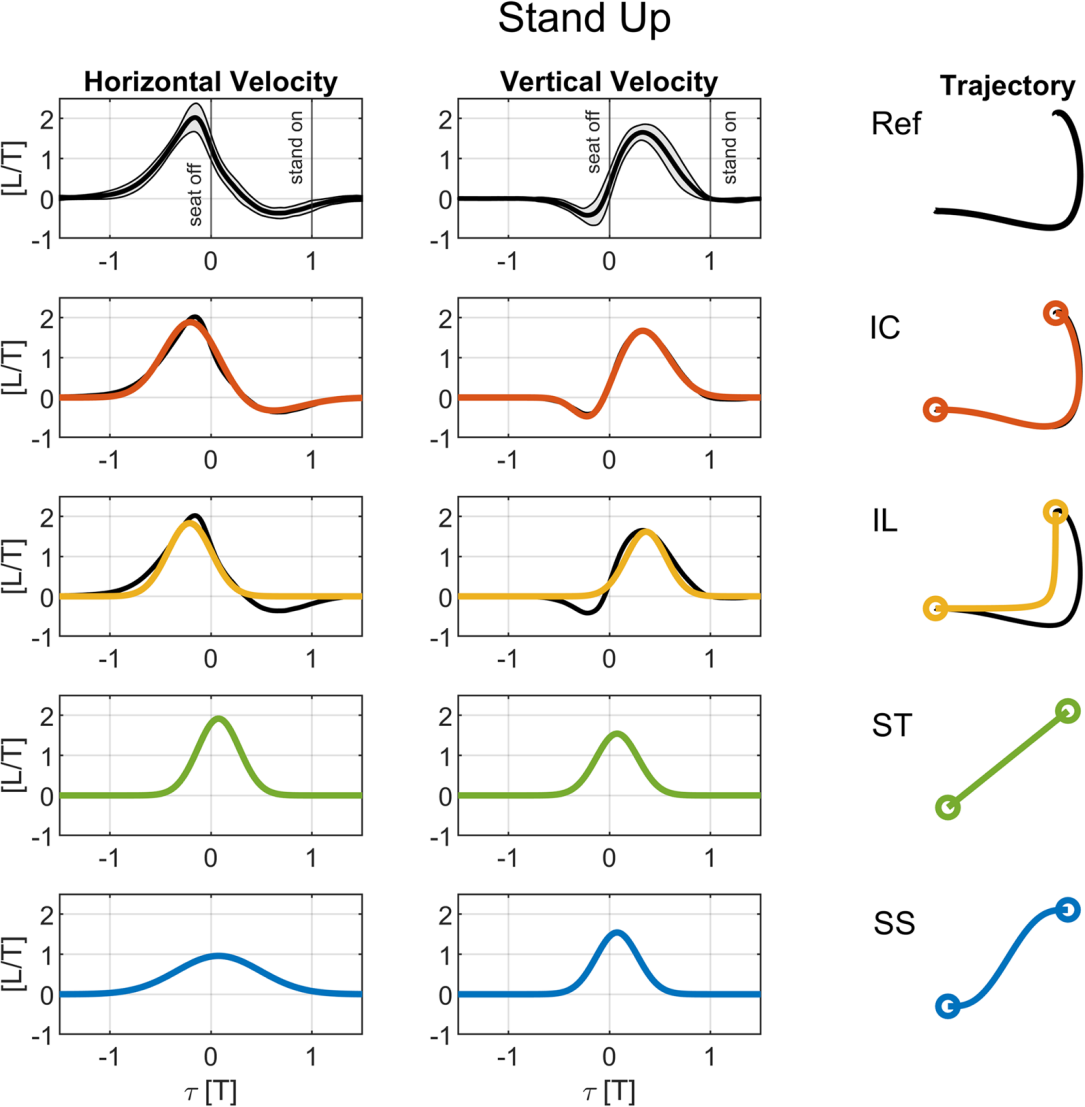
Horizontal and vertical velocity profiles and resulting trajectories for standing up. The chart on the top row shows experimental average shoulder velocity profiles and trajectory shape for the reference unassisted standing up data (*Ref*, average $\pm \sigma$ in black and gray shade). The more curved trajectories C-shape (*IC*) and L-shape (*IL*) ($2^{nd}$ and $3^{rd}$ rows) are compared to the reference curves, which they approximate. The straighter trajectories Straight (*ST*), and S-shape (*SS*) ($4^{th}$ and $5^{th}$ rows) are derived from the *IL*. All trajectories are normalized by thigh length *L* and duration *T*, and the normalized time $\tau$ is defined as in Eq. 5. The *seat off* event occurs at $\tau =0$, and the *stand on* event occurs at $\tau =1$ in the experimental, reference data *Ref*

**Fig. 3:**
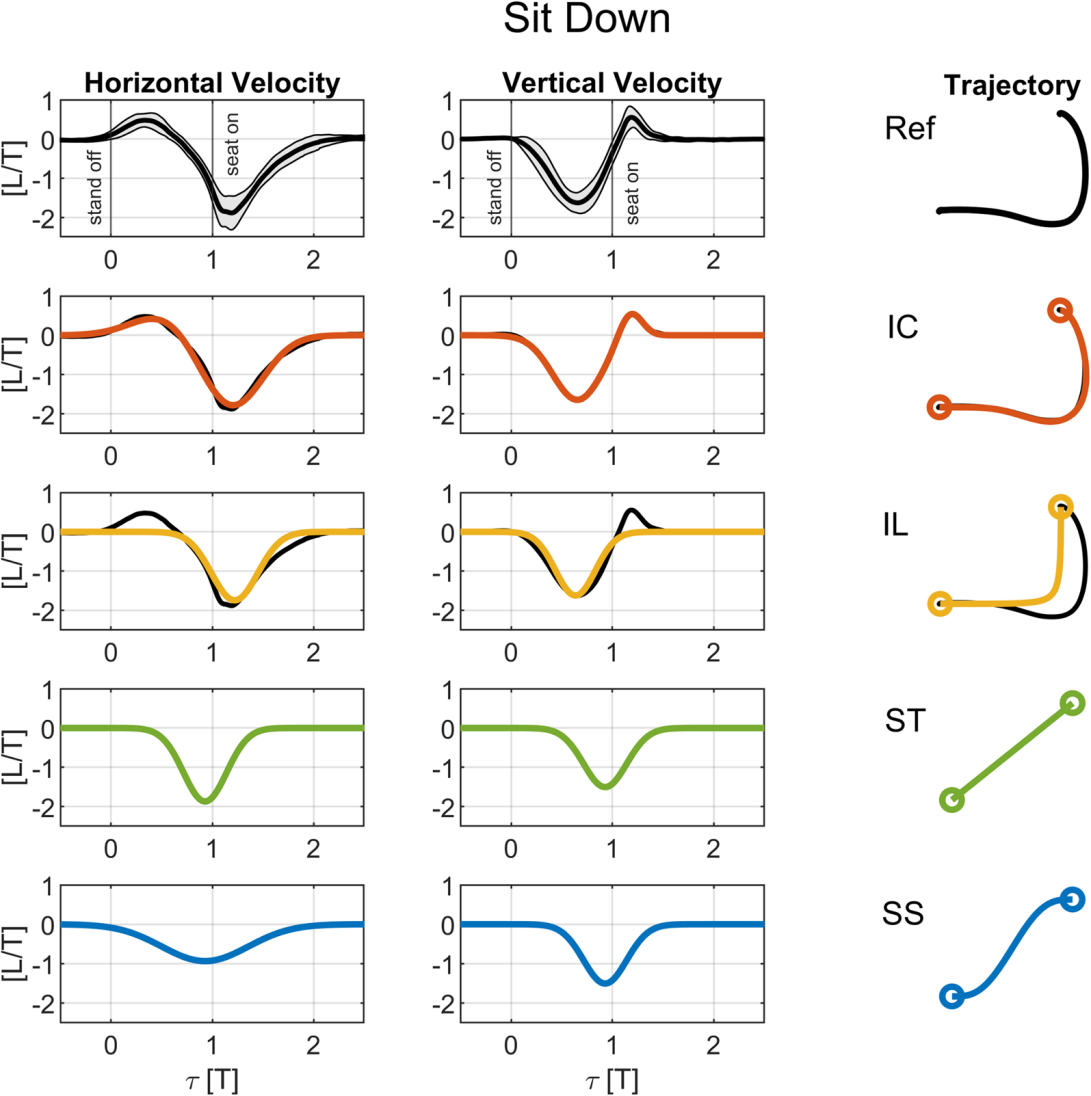
Horizontal and vertical velocity profiles and resulting trajectories for sitting down. The charts on the top row show experimental average shoulder velocity profiles and trajectory shape for the reference unassisted sitting down data (*Ref*, average $\pm \sigma$ in black and gray shade). The more curved trajectories C-shape (*IC*) and L-shape (*IL*) ($2^{nd}$ and $3^{rd}$ rows) are compared to the reference curves, which they approximate. The straighter trajectories Straight (*ST*), and S-shape (*SS*) ($4^{th}$ and $5^{th}$ rows) are derived from the *IL*. All trajectories are normalized by thigh length *L* and duration *T*, and the normalized time $\tau$ is defined as in Eq. 5. The *stand off* event occurs at $\tau =0$, and the *seat on* event occurs at $\tau =1$ in the experimental, reference data *Ref*

The reference shoulder trajectories, which inspire the c-shaped trajectories described in the following section, are assessed from a previously recorded dataset of unassisted sit-to-stand and stand-to-sit motions in 10 younger adults (28±5 years; [37]). The participants stood up and sat down five times at their own comfortable pace, with two seconds of rest in between each motion, while their full body motion was tracked and automatically segmented based on a clustering algorithm [37]. We selected the left acromion marker of each individual for analysis. The data was filtered (zero-lag Butterworth, 6 Hz) and normalized to thigh length *L*, taken as the average distance over time between the markers on the *greater trochanter* and the *femoral lateral epicondyle*. The time for each repetition was divided (normalized) by the duration *T*, which ranges from *seat off*, when the participant’s buttocks leave the chair, to *stand on*, when the participant achieves full, quiet standing, or from *stand off*, when the participant starts moving to sit down, to *seat on*, when the participant touches the chair for sitting. To calculate normalized velocity profiles, the first derivative of the normalized position profile was computed by finite differences in the horizontal and vertical directions separately. The mean and standard deviations of the normalized velocities over all 5 movement repetitions and ten participants were computed for standing up and sitting down, see the reference velocity profiles and corresponding trajectories on the top charts in Figs. 2 and 3. Note that due to time normalization, for standing up *seat off* occurs at $\tau = 0$ and *stand on* at $\tau = 1$, and for sitting down *stand off* occurs at $\tau = 0$ and *seat on* at $\tau = 1$ for the reference data.

#### C-shape trajectory (*IC*)

We sought to parameterize the shoulder velocity profiles, and they can be well approximated by a pair of Gaussian-like functions as

1 $$\begin{aligned} \tilde{v}_{i,j}(\tau ) = a_{1,i,j} e^{- \left( \tau -b_{1,i,j}\right) / c_{1,i,j}} + a_{2,i,j} e^{- \left( \tau -b_{2,i,j}\right) / c_{2,i,j}}, \end{aligned}$$

where $\tau$ is the normalized time, *i* the standing up ($i=u$) or sitting down ($i=d$) movement, and *j* the horizontal ($j=h$) or vertical ($j=v$) directions. The 6 parameters (coefficients *a*, *b*, and *c*) were determined by solving optimal curve fitting problems using the interior-point algorithm in the MATLAB nonlinear optimization function *fmincon* subject to constraints on the horizontal and vertical displacements of the wrist that ensure they are equal to the corresponding average measured displacements. As this profile leads to an inverted C-shaped trajectory, it is referred to in this paper as *IC*. Note that this simple parameterization results in a good approximation of the shoulder velocity profiles and resulting shoulder trajectory for standing and sitting, as evidenced by the charts in Figs. 2 and 3. The coefficients for the velocity profiles of this and the following trajectories are provided in Tables 1-4 of the Appendix A, according to Eq. 1.

#### L-shape trajectory (*IL*)

The *IC* trajectory proceeds from the initial to the final position non monotonically. For standing up, for instance, the handles move downwards before going upwards, and forwards beyond the final position. This could be perceived as counterintuitive and uncomfortable by users, and would require inverting the actuators’ direction. Therefore, an alternative trajectory was generated by fitting the reference trajectory with a single Gaussian-like curve as

2 $$\begin{aligned} \tilde{v}_{i,j,IL}(\tau ) = a_{i,j,IL} e^{- \left( \tau -b_{i,j,IL}\right) / c_{i,j,IL}}, \end{aligned}$$

subject to the same displacement constraints. This effectively results in a monotonic motion of the handles with strictly positive (standing) or negative (sitting) horizontal and vertical velocities and an inverted L-shape trajectory, referred to as *IL* (see charts in Figs. 2 and 3).

#### Straight trajectory (*ST*)

From the velocity profiles of *IL*, we generated two additional trajectories providing more direct paths from the initial to the final position (see Figs. 2 and 3), whose shapes are closer to some of the ones investigated in the literature [24, 38]. The first is a straight trajectory connecting initial and final handle positions, referred to as *ST*. We obtained this by averaging the *b* and *c* values obtained for the horizontal and vertical directions for the *IL* trajectory as

3 $$\begin{aligned} \tilde{v}_{i,j,ST}(\tau ) = a_{i,j,ST} e^{- \left( \tau -b_{i,j,ST}\right) / c_{i,j,ST}}, \end{aligned}$$

with

$$\begin{aligned} b_{i,h,ST} = b_{i,v,ST}= & (b_{i,h,IL} + b_{i,v,IL}) / 2 , \\ c_{i,h,ST} = c_{i,v,ST}= & (c_{i,h,IL} + c_{i,v,IL}) / 2 . \end{aligned}$$

The coefficients $a_{i,j,ST}$ were adjusted to satisfy the horizontal and vertical displacement constraints.

#### S-shape trajectory (*SS*)

The last alternative trajectory is an s-shape trajectory (see Figs. 2 and 3), referred to as *SS*, designed to provide a smoother transfer by halving the peak horizontal velocity as

4 $$\begin{aligned} \tilde{v}_{i,j,SS}(\tau ) = a_{i,j,SS} e^{- \left( \tau -b_{i,j,SS}\right) / c_{i,j,SS}}, \end{aligned}$$

with all coefficients reproducing the ones for *ST*, except for

$$\begin{aligned} a_{i,h,SS}= & a_{i,h,ST} / 2, \\ c_{i,h,SS}= & 2 c_{i,h,ST} . \end{aligned}$$

The $c_{i,h,SS}$ coefficients are doubled to satisfy the horizontal displacement constraints. Based on their general shape, the *ST* and *SS* will be referred to as the “straighter trajectories” as opposed to *IC* and *IL* as the “curved trajectories”.

#### Scaling of trajectories

The velocity profiles for these trajectories $\tilde{v}_{i,j}$ (Eqs. 1–4) can be scaled to the desired STS duration *T* and user’s anthropometry, so that the total vertical displacement of the handle corresponds to the measured difference between the wrist height when standing $h_{st}$ and the wrist height when sitting $h_{si}$ as

5 $$\begin{aligned} v_{i,j}(t) = \frac{h_{st}-h_{si}}{|\Delta \tilde{h}|} \, \tilde{v}_{i,j}(\tau = t/T), \end{aligned}$$

where

$$\begin{aligned} \Delta \tilde{h} = \int _{\tau _0}^{\tau _f} \tilde{v}_{i,v} \, d\tau \end{aligned}$$

is the normalized vertical displacement of the trajectory, with $\tau _0 = -1.5$ and $\tau _f = 1.5$ for standing up, and $\tau _0 = -0.5$ and $\tau _f = 2.5$ for sitting down.

#### Static handle condition (*SH*)

In our study design, the moving-handle assistance provided through the previously introduced trajectories will be compared to static handle assistance similar to that provided by a conventional passive rollator. In this baseline condition, the handles are moved to the same final position as the other trajectories (in front of the participants) before the start of the trial. The handles remain in this position until all the standing up and sitting down repetitions are completed by the participants, as will be explained in more detail further on in “Experimental protocol” section.

### Experimental set-up

To assess our handle trajectories, we recruited 15 young able-bodied adults (3 female; $27.5 \pm 4.9$ yrs.; $69.2 \pm 10.0$ kg; $1.74 \pm 0.05$ m; Table 5 in Appendix B). Exclusion criteria were any verbally self-declared neurological, cardiovascular, metabolic, psychiatric problems, or (sport) injuries that could interfere with the planned tasks. The protocol was approved by the Institutional Review Board of the Medical Faculty of Heidelberg University (protocol S-654/2019), and all participants provided written informed consent.

The age, gender, height, mass, wrist (ulna head) height while standing, wrist height while sitting, and horizontal distance between the wrists were collected with arms relaxed hanging down. A stool was connected to the rear force plate at a predefined position with velcro tape and its height was adjusted so that the participant’s thigh was horizontal and shank was vertical. The participant was asked to sit on the stool with the feet on the front force plate, see Fig. 1, with the thigh horizontal and the shank vertical.

Full-body motion capture data was collected using a passive optical motion capture system (10 cameras, Qualisys, Gothenburg, Sweden) at 150 Hz. An adjusted version of the IOR full body marker set [39, 40] was used consisting of 49 (14 mm) markers, with a reduced number of markers on the trunk to reconstruct pelvis and trunk segments, and additional iliac crest and greater trochanter markers to ensure tracking throughout the STS motion cycle. Ground reaction forces were collected simultaneously using two ground-embedded force plates at 900 Hz (Bertec, Columbus, OH, USA). Handle forces and moments were collected by two 6-axis load cells (FT300, Robotiq, Lévis, QC, Canada) at 100 Hz. The velocity of each axis of the linear gantry assembly is derived from the rotor position transducers of the respective motors, and the mechanical transmission ratio. This corresponds to the horizontal and vertical velocity components of the handle and is provided at 1000 Hz. All raw data were low-pass filtered with a bidirectional, zero-lag, fourth-order Butterworth filter with a cut-off frequency of 6 Hz in Matlab (Mathworks, Natick, USA).

### Experimental protocol

The experimental session proceeded in 4 blocks: 01) Collection of static calibration trial and unassisted STS; 02) Familiarization; 03) Evaluation of trajectories; 04) Evaluation of velocities.

In block 01, after the collections of a static calibration trial where participants stand on the front force plate in a T-pose for 5 s, participants performed 5 repetitions of unassisted standing up and sitting down at a comfortable velocity, with approximately 2 s between the end of one movement and the beginning of the subsequent one to help motion segmentation. After completion of block 01, the participant rested for about 10 minutes while the *RAS* device was installed. The two sides of the device are placed in parallel, symmetrically to the middle line of the force plates, and so that the distance between the bilateral handles corresponds to the measured horizontal distance between the participant’s wrists plus 100 mm to ensure sufficient clearance to the stool. In its initial position, each handle is laterally aligned with the stool at a height corresponding to the previously measured height of the wrist while sitting. For safety, the participants are asked to remain at all times within the yellow stripes (Fig. 1a), except for the arms interacting with the handles. The velocity profiles for all trajectories are scaled to the corresponding STS duration *T* and measured difference between the wrist height when standing and the wrist height when sitting according to Eq. 5.

Block 02 was designed to familiarize the participant with the static handle assistance condition *SH* and the 4 assistance trajectory conditions (*IC*, *IL*, *ST*, and *SS*) for $T=2$ s, which is a duration considered subjectively comfortable in previous tests (with *T* values ranging from 1.0 to 3.5 s, unpublished), and *T* defined as in “Reference trajectories” section. The participants performed 2 repetitions of standing up and sitting down for each one of the conditions in a randomized order, without the collection of data. To indicate the beginning of the trial, 3 beeps followed by a higher-pitch beep are generated. After the completion of the first standing up, the subsequent start of sitting down or standing up movements are indicated by single beeps (after 8 s). For the *SH* condition, the handles are moved to the final position before the start of the trial, and the participants are instructed to stand up or sit down every time a beep is generated, similarly to the procedure with moving handle conditions.

In evaluation Block 03, the participants performed 5 consecutive repetitions of standing up and sitting down for each of the 5 assistance conditions at $T=2$ s in a randomized order. The procedure within a trial is identical to the one in Block 02. After each trial and condition is completed, the participant is asked to provide a subjective evaluation on the level of perceived support and stability on a 5-point scale, as well as on the perceived velocity on a 3-point scale, for standing up and sitting down (see detailed questionnaire in Appendix C).

The velocity Block 04 was designed to test the effect of the overall velocity of the handle on the assistance performance. For this purpose, 4 different handle velocities were tested for the condition *SS* in a similar procedure to Black 03. To reduce the number of necessary trials, only the condition *SS* was tested, which was the trajectory subjectively considered more favorable in previous pilot tests. The normalized velocity profiles were scaled according to Eq. 5 with values *T* ranging from $T=1.5$ s (highest velocity) to $T=3.0$ s (lowest velocity) in steps of 0.5 s. Although the average reference unassisted data time for standing up was $\overline{T}=0.91$ s (from *seat off* to *stand on*) and $\overline{T}=1.02$ s for sitting down (from *stand off* to *seat on*), the maximal velocity subjectively considered safe and comfortable in pilot experiments with moving handles was the one corresponding to $T=1.5$ s. Durations higher than $T=3.0$ s were considered too long in pilot experiments.

### Data evaluation

The time events of *seat off* and *seat on* were identified for each repetition using a 10 N threshold on the vertical force recorded by the force plate under the stool. 3D kinematics and body COM position were calculated using the IOR full-body model, adjusted to have separate pelvis and trunk segments, and anthropometric properties from [41] in Visual3D (C-motion, Inc., Germantown, MD, USA). Sagittal knee and hip joint moments were calculated as follows. First, the knee and hip sagittal plane moments due to gravity and inertial effects were computed for each leg by bottom-up inverse dynamics in Visual3D, without considering the ground reaction forces. Second, as both feet are placed on the same force plate, the contribution of the ground reaction forces to the joint moments was computed in Matlab, assuming bilateral symmetry and splitting the vertical and anterior-posterior GRF components equally between both feet. Finally, to compute total hip and knee moments, the moments due to inertial and gravitation effects computed in Visual3D and the moments due to the GRF computed in Matlab were summed up. The reported values correspond to the average of the left and right joint moments. The different assistance conditions (trajectories and static handle) were compared in terms of the lower limb effort, the magnitude of the support provided by the handles, and subjective perception.

Lower limb effort was quantified as the peak of sagittal hip and knee extension moments, averaged over the *m* valid repetitions, normalized by the $i^{th}$ participant’s height ($H_i$) and total body weight ($BW_i$), as

6 $$\begin{aligned} \overline{M}_{h,max,i}= & \frac{1}{(BW_i H_i)} \frac{1}{m} \sum _{j=1}^{m} \max (M_{h,i,j}) , \end{aligned}$$

7 $$\begin{aligned} \overline{M}_{k,max,i}= & \frac{1}{(BW_i H_i)} \frac{1}{m} \sum _{j=1}^{m} \max (M_{k,i,j}) , \end{aligned}$$

where $M_{h,i,j}$ and $M_{k,i,j}$ are the average of the left and right hip and knee extension moments (positive in extension) of the $i^{th}$ participant in the $j^{th}$ repetition of standing up or sitting down in the corresponding assistance condition. Repetitions were considered invalid in three instances: *i*) when the participant stepped outside of the front force plate, even if only partially, which was inspected visually during the experiment; *ii*) when it was not possible to fully reconstruct the kinematics of any body segment in Visual3D due to marker occlusions; or *iii*) when data transmission disruptions from the load cell to the acquisition computer where identified.

Support magnitude provided by both handles was quantified as the maximal total bilateral vertical force, the maximal total horizontal (anterior-posterior) force, and the total work exerted by both handles. The total vertical and horizontal forces are normalized by the participant’s weight as

8 $$\begin{aligned} \overline{F}_{v,max,i}= & \frac{1}{BW_i} \frac{1}{m} \sum _{j=1}^{m} \max (F_{v,i,j}) , \end{aligned}$$

9 $$\begin{aligned} \overline{F}_{h,max,i}= & \frac{1}{BW_i} \frac{1}{m} \sum _{j=1}^{m} \max (|F_{h,i,j}|) , \end{aligned}$$

where $F_{v,i,j}$ and $F_{h,i,j}$ are the total vertical and horizontal force components, respectively, applied by both handles on the $i^{th}$ participant at the $j^{th}$ valid repetition of standing up or sitting down in the corresponding assistance condition, with upward force and forward force (pulling of the handle) applied to the hands as positive.

The total work exerted by the handles is normalized by the gravitational potential energy difference between sitting and standing as

10 $$\begin{aligned} \overline{W}_i = \frac{1}{BW_i |\Delta z_{G,i}|} \frac{1}{m} \sum _{j=1}^{m} W_{i,j} \, , \end{aligned}$$

where $W_{i,j}$ is the total mechanical work exerted by both handles to the $i^{th}$ participant at the $j^{th}$ valid repetition of standing up or sitting down in the corresponding assistance condition. $\Delta z_{G,i}$ is the vertical displacement of the center of mass of the $i^{th}$ participant, which is nearly the same for all the conditions. To ensure the same normalization factor for each participant over all conditions, the vertical displacement of the center of mass $\Delta z_{G,i}$ is computed as the average over the repetitions in the unassisted condition (*UN*) of participant *i*. The total work exerted by the handles is computed as

11 $$\begin{aligned} W_{i,j} = \int _{t_0}^{t_f} \vec {F}_{i,j} \cdot \vec {v}_{i,j} \, dt \, , \end{aligned}$$

where $\vec {F}_{i,j}$ is the total force applied by the handles to the hands, $\vec {v}_{i,j}$ is the handle velocity, and $t_0$ and $t_f$ are the initial and final times.

As many of the evaluation metrics are not normally distributed according to the Shapiro-Wilk normality test, we conducted non-parametric Friedman tests on the five outcome metrics (leg effort: peak knee and hip extension; support: peak vertical and anterior-posterior force, and total handles’ work). Significance was set at $p=0.05$. For the assistance comparison (block 3), we performed a single-factor (assistance type) analysis with 5 conditions (*SH*, *IC*, *IL*, *ST*, and *SS*). Although data on unassisted standing up and sitting down were collected, they were not included in the statistical analysis because the order was not randomized, as they were always collected at the beginning of the experiment in block 1. Post-hoc analysis was conducted using pairwise Wilcoxon signed-rank tests with significant values adjusted by the Bonferroni correction for multiple tests. For the velocity comparison (block 4), we performed a single-factor (velocity) analysis with 4 conditions (*T15*, *T20*, *T25*, *T30*). The same post-hoc analysis was performed. All statistical computations were performed in SPSS Statistics (IBM, Armonk, NY, USA).

## Results

**Fig. 4:**
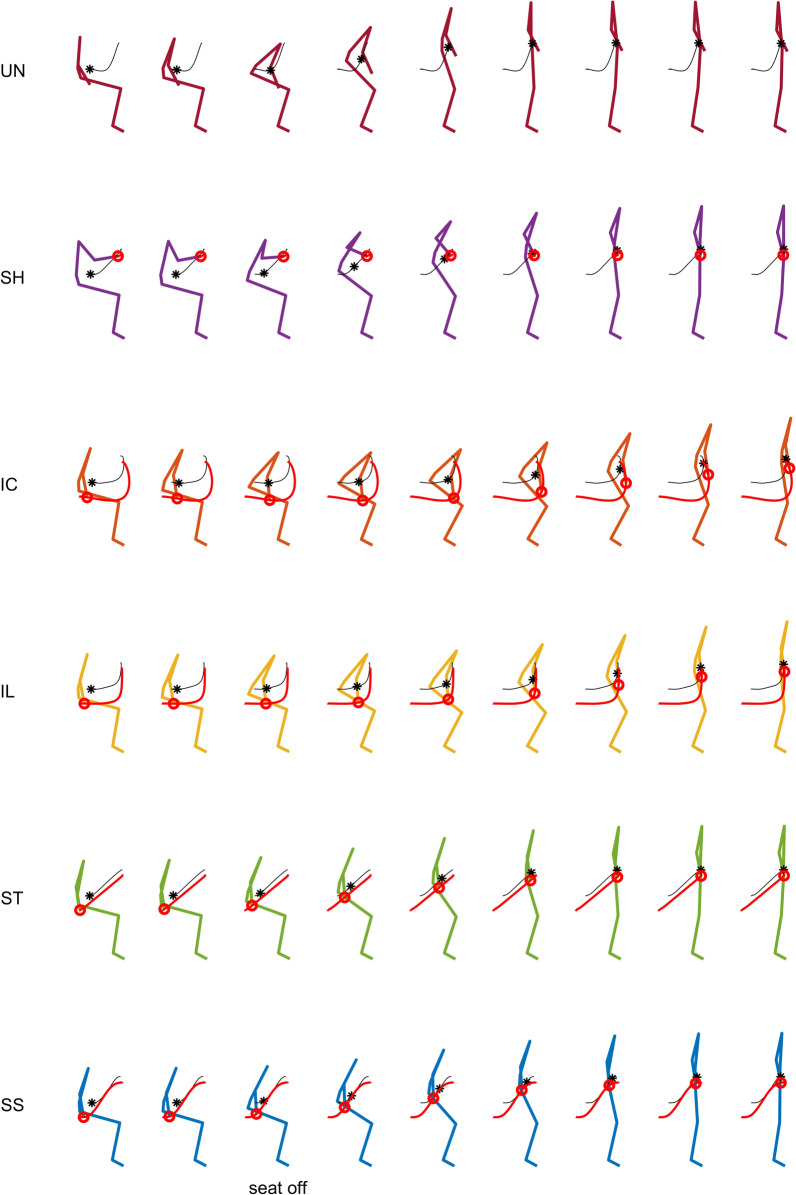
Stick-figure snapshots of average standing up sagittal kinematics for unassisted STS (*UN*), STS assisted with static handles (*SH*), and STS supported by the four types of assistance trajectories (*IC*, *IL*, *ST*, *SS*), indicated by the red solid lines. Snapshots are 0.3 s apart, and the third snapshot from the left represents the *seat off* instant. The *red circle* indicates the wrist position, the *black star* the whole body’s center of mass (CoM), and the *black solid line* the CoM trajectory.

**Fig. 5:**
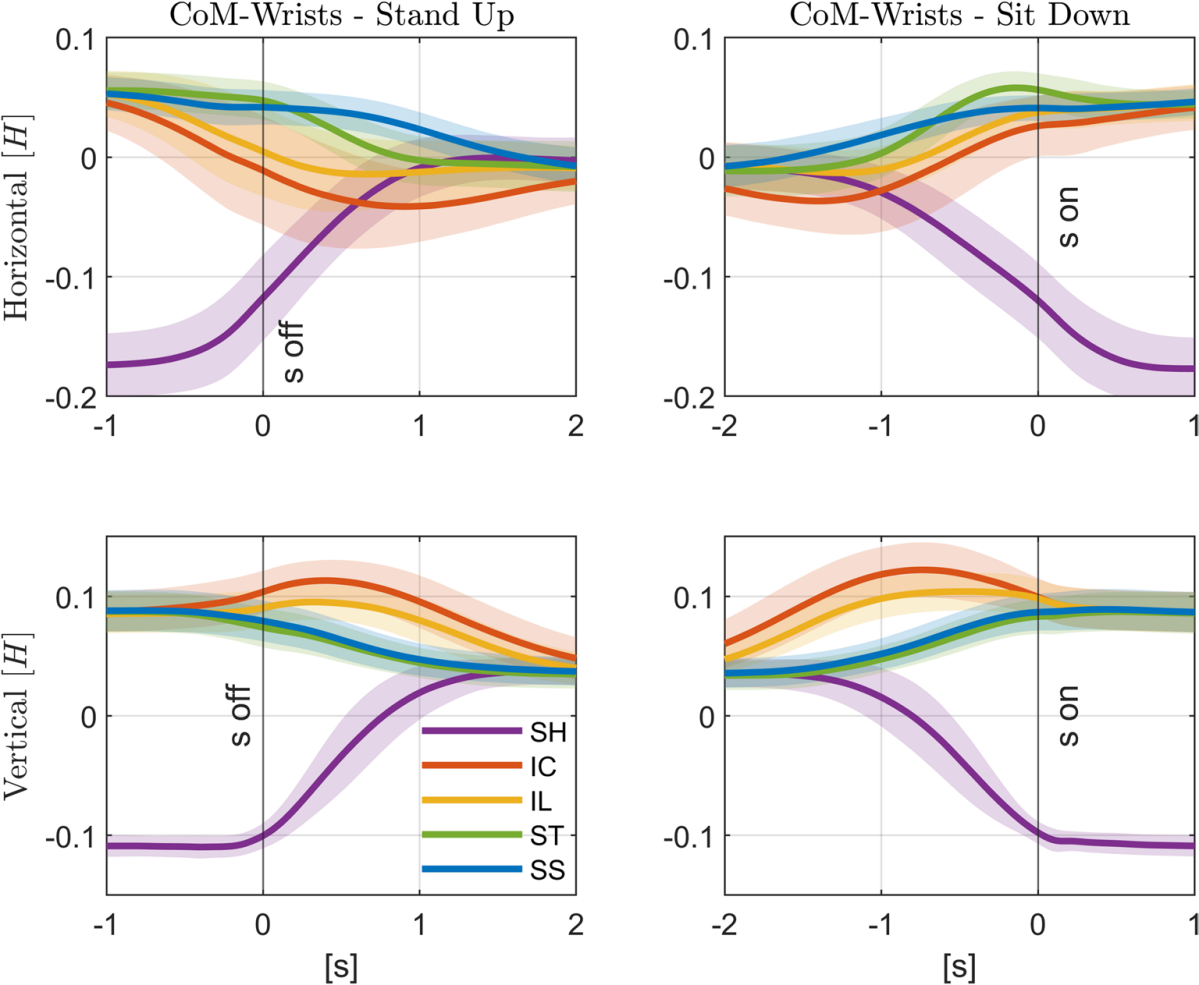
Relative position of COM with respect to wrists over time. Average difference between the position of the body’s COM and the right and left wrists in the horizontal (*top*) and vertical (*bottom*) directions for standing up (*left*) and sitting down (*right*), where positive values correspond to COM in front and above the wrists, respectively. The different conditions (STS assisted with fixed handles (*SH*), and assistance trajectories (*IC*, *IL*, *ST*, and *SS*)) are shown in different colors with ± one standard deviation around the average represented by the corresponding *shaded areas*. Time zero corresponds to the *seat off* instant (“s off”) for standing up, and to the *seat on* instant (“s on”) for sitting down. Note: only valid repetitions were considered in the analysis (as described in “Data evaluation” section)

**Fig. 6:**
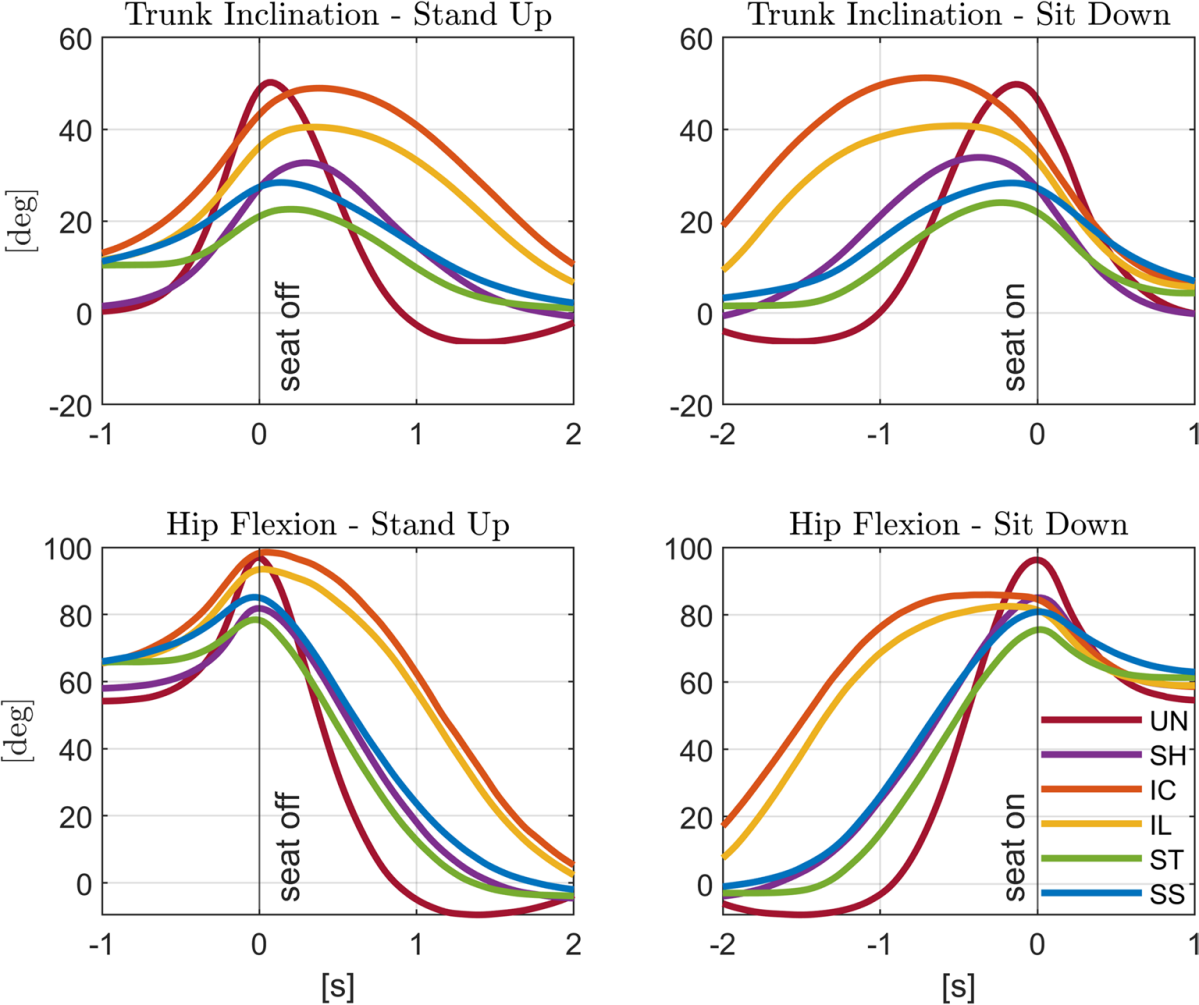
Trunk inclination and hip flexion angle. Average trunk inclination angle with respect to the vertical (*top*, positive for forward bending), and hip flexion angle (*bottom*, thigh with respect to pelvis, positive for flexion), for standing up (*left*) and sitting down (*right*). The different conditions (unassisted STS (*UN*), STS assisted with fixed handles (*SH*), and assistance trajectories (*IC*, *IL*, *ST*, and *SS*)) are shown in different colors. Time zero corresponds to the *seat off* instant for standing up, and to the *seat on* instant for sitting down

For context of interpreting the effect of the handle support, we first describe changes in the body posture with different trajectories. The body center of mass (COM) changes its trajectory during the static vs. moving handle conditions during both standing up and sitting down (Fig. 4). While the COM stays behind and below the handle in the *SH*, it is above and approximately vertically aligned during the curved trajectories (*IC* and *IL*), and above and slightly in front during straighter trajectories (*ST* and *SS*), as can be seen in Fig. 5. This corresponds with a change in upper body kinematics from the curved trajectories to the straighter ones. The assistance with the curved *IC* and *IL* have overall kinematics resembling that of the unassisted standing up, and requires pronounced trunk inclination and hip flexion as the handles move forward first before moving upwards, refer to Fig. 6. The straighter trajectories show less trunk inclination, less hip flexion, and an earlier rising of the body from the stool. As required, the shoulder and the elbow joints remain nearly vertically aligned with the handle during all moving handle conditions. This is likely to reduce the arm joint moments required to transmit the large vertical forces applied by the handles to the upper body.

**Fig. 7:**
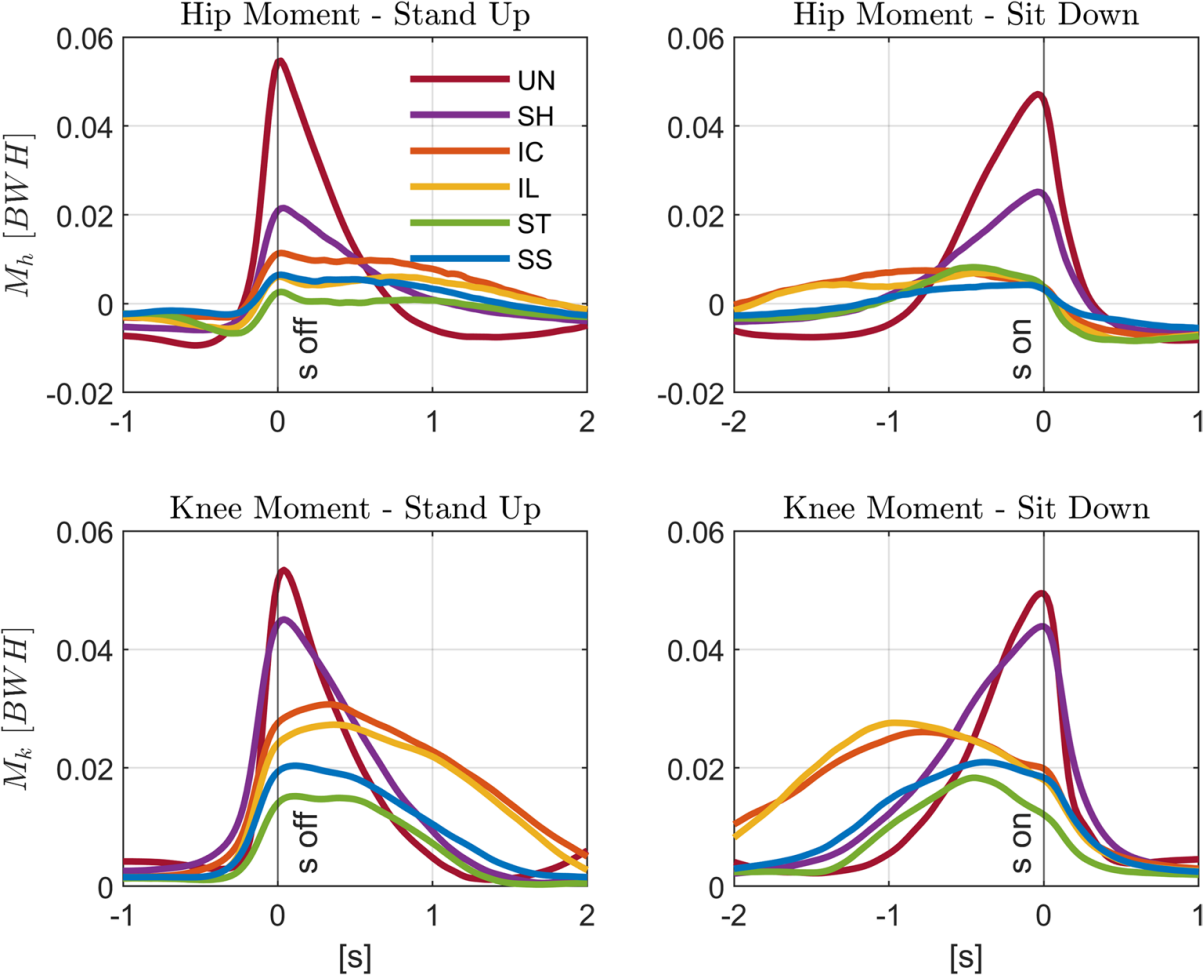
Effect of handle assistance on leg loading. Group-average hip ($M_{h}$, *top*) and knee ($M_{k}$, *bottom*) joint extension moments in the sagittal plane normalized by body weight (*BW*) and height (*H*) for both standing up (*left*) and sitting down (*right*). The different conditions (unassisted STS (*UN*), STS assisted with fixed handles (*SH*), and assistance trajectories (*IC*, *IL*, *ST*, and *SS*)) are shown in different colors. Time zero corresponds to the *seat off* instant (“s off”) for standing up, and the *seat on* instant (“s on”) for sitting down

**Fig. 8:**
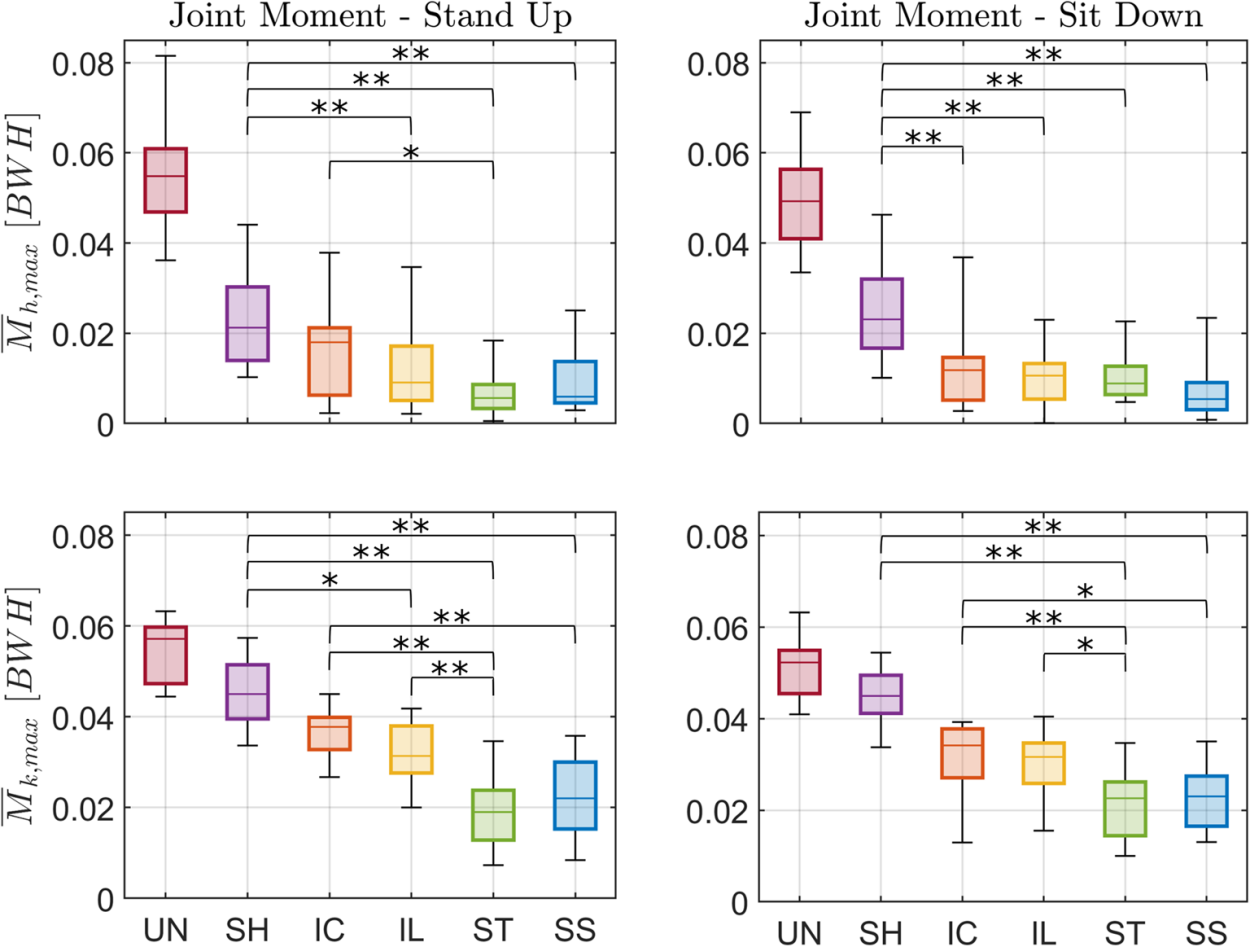
Effect of handle assistance on peak leg loading. Boxplots of median and interquartiles (25 and 75%) of peak hip ($\overline{M}_{h,max}$, top) and knee ($\overline{M}_{k,max}$, bottom) joint extension moments in the sagittal plane normalized by body weight (*BW*) and height (*H*) for both standing up (*left*) and sitting down (*right*). The different conditions (unassisted STS (*UN*), STS assisted with fixed handles (*SH*), and assistance trajectories (*IC*, *IL*, *ST*, and *SS*)) are shown in different colors. Significant differences are indicated by * for $p < 0.05$ or by ** for $p < 0.01$. *UN* was not included in the statistical analysis because its order was not randomized in the experiments

Lower limb joint loading is substantially reduced in the moving handle conditions compared to unassisted STS and to the static handle (*SH*) during both standing up and sitting down (Figs. 7 and 8, and Tables 6 and 7 in Appendix D). Compared to standing up with static handle support (*SH*), the median peak hip moment was reduced by 57% for *IL* ($p < 0.01$), 74% for *ST* ($p < 0.01$), and 72% for *SS* ($p < 0.01$). Compared to sitting down with static handle support (*SH*), the median peak hip moment was reduced by 49% for *IC* ($p < 0.01$), 54% for *IL* ($p < 0.01$), 61% for *ST* ($p < 0.01$), and 77% for *SS* ($p < 0.01$). There is a tendency for a further reduction of hip moment by the straighter trajectories (*ST*, *SS*) compared to the curved ones (*IC*, *IL*), but differences were mostly not statistically significant.

When comparing to the unassisted STS, the reduction of peak knee moments is also substantial, although less pronounced than the one achieved for the hip joint moment. Note that the different assistance conditions change the lower body joints loading distribution to higher peak knee with respect to hip moments compared to an equal knee and hip contribution during unassisted standing up. Reductions achieved with moving handles were substantial compared to static handles (*SH*), particularly for straighter handle trajectories in both standing up and sitting down. The median peak knee moment was reduced by 30% for *IL* ($p < 0.05$), 58% for *ST* ($p < 0.01$), and 51% for *SS* ($p < 0.01$). For sitting down, the median peak knee moment was reduced by 50% for *ST* ($p < 0.01$), and 49% for *SS* ($p < 0.01$) compared to *SH*. The straighter trajectories, particularly the *ST*, led to lower peak knee moments compared to the curved trajectories (*IC*, *IL*), with reductions of 50% (*IC*-*ST*, $p < 0.01$) and 39% (*IL*-*ST*, $p < 0.01$) for standing up, and of 34% (*IC*-*ST*, $p < 0.01$) and 28% (*IL*-*ST*, $p < 0.05$) for sitting down.

**Fig. 9:**
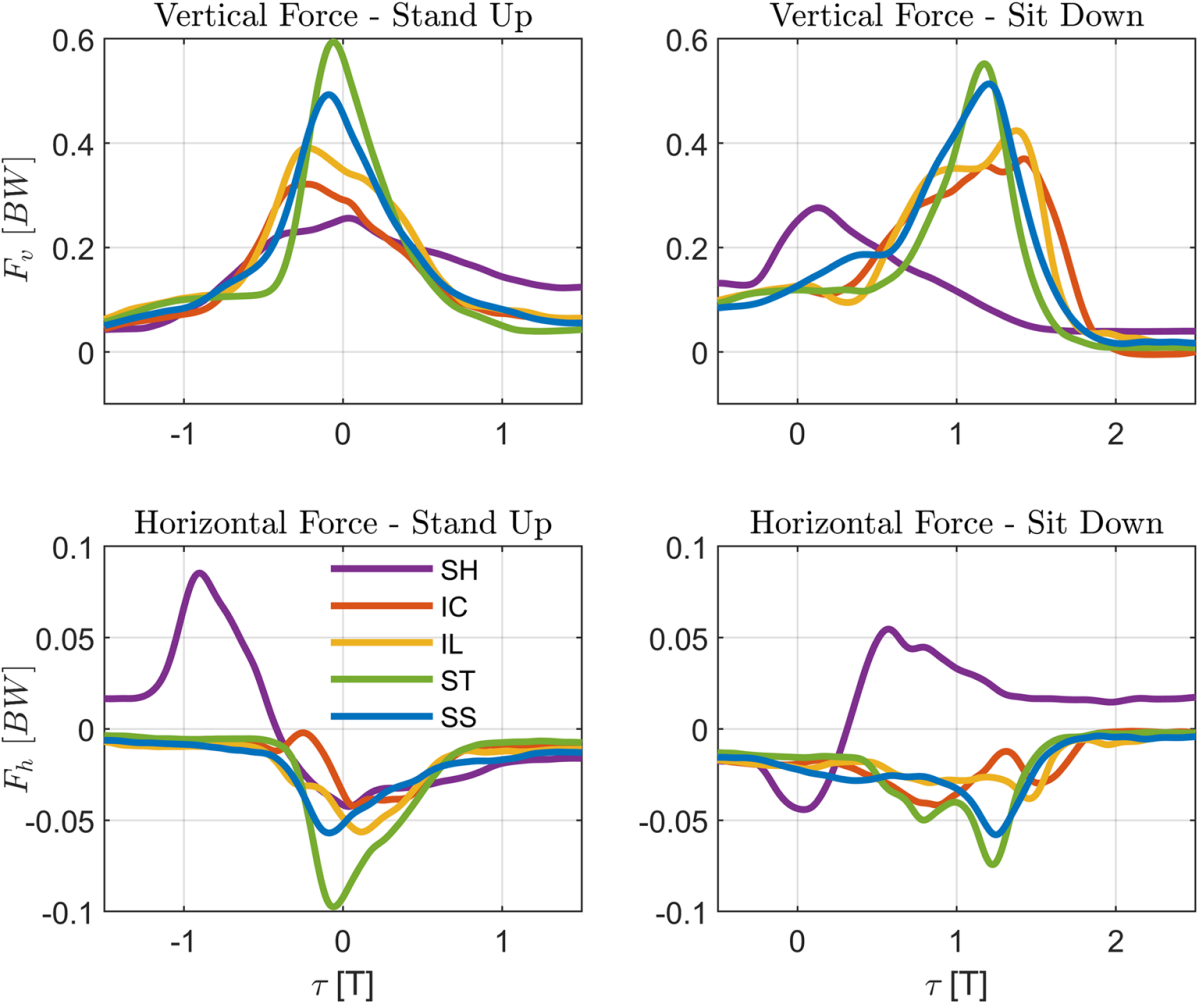
Effect of handle assistance on handle forces. The group-average vertical (positive upwards, *top*) and horizontal (positive for pulling, *bottom*) forces measured for both handles are normalized by body weight (*BW*) for standing up (*left*) and sitting down (*right*). Handle trajectories with fixed handles (*SH*), and assistance trajectories (*IC*, *IL*, *ST*, *SS*) are shown in different colors. The normalized time $\tau$ is defined as in Eq. 5, according to the motion of the handles as in Figs. 2 and 3, with the duration $T=2$ s. The *SH* force trajectories were shifted by $T/2 = 1$ s to facilitate comparison and to reflect the fact that, while handles start moving after the one-second long beep in the moving-handle conditions, the participants start the movement as soon as the beep starts in the *SH* condition. *seat off* and *seat on* events not indicated because the handle force data was not rigorously synchronized with force plate data, used to identify them

**Fig. 10:**
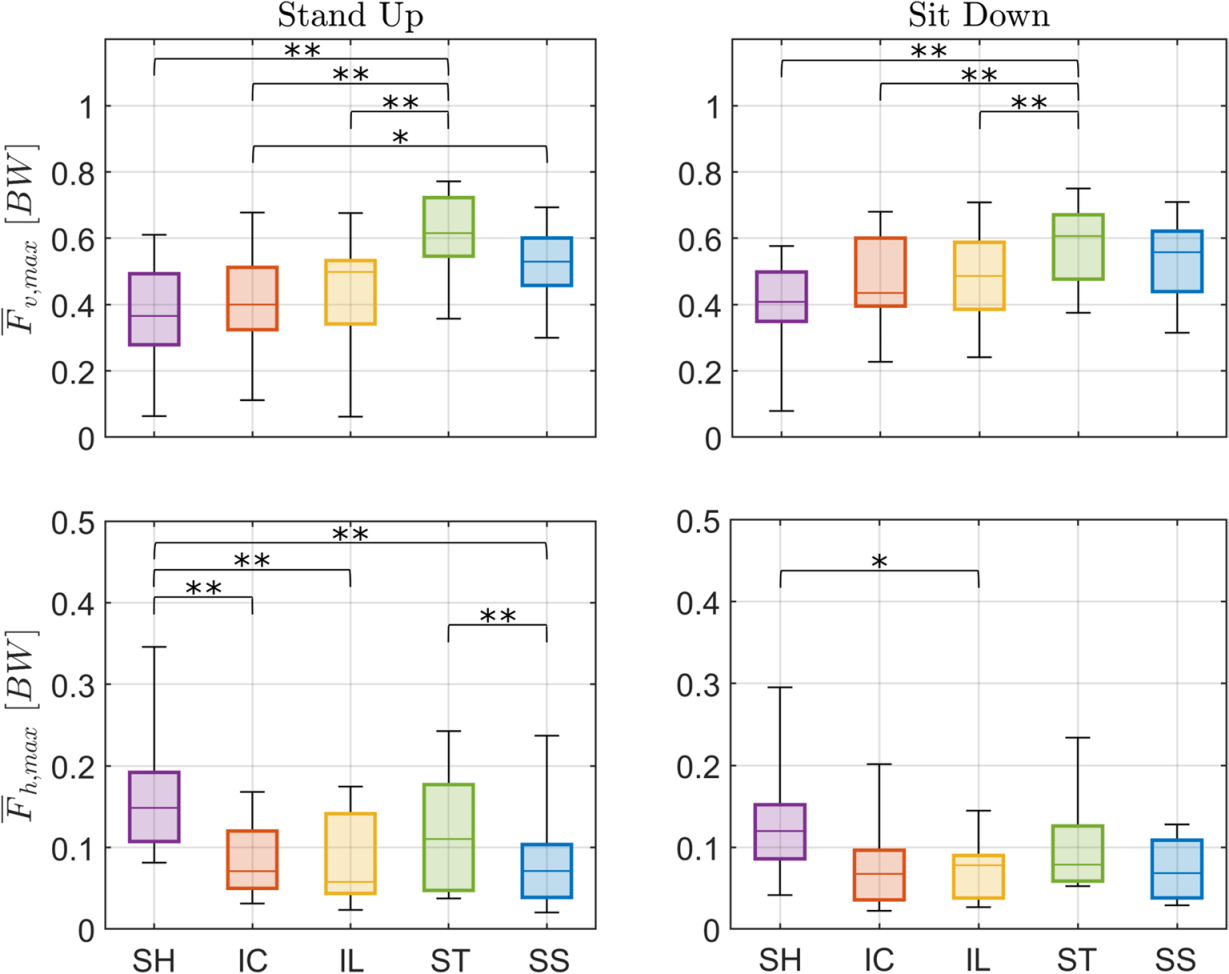
Effect of handle assistance on peak handle forces. Boxplots of median and interquartiles (25 and 75%) of evaluation metrics peak vertical ($\overline{F}_{v,max}$: Eq. 8, *top*) and horizontal ($\overline{F}_{h,max}$: Eq. 9, *bottom*) handle forces are shown for standing up (*left*) and sitting down (*right*). The different conditions (STS assisted with fixed handles (*SH*), and assistance trajectories (*IC*, *IL*, *ST*, and *SS*)) are shown in different colors. Statistically significant differences are indicated by * for $p < 0.05$ or by ** for $p < 0.01$

The provided moving-handle support changed the interaction, with lower leaning vertical forces and larger horizontal pulling forces in *SH* being replaced by larger vertical forces and lower pushing horizontal forces for the moving-handle assistance (Figs. 9 and 10, and Tables 6 and 7 in Appendix D). Between the moving handle conditions, there is a tendency for straighter trajectories, particularly *ST*, to elicit significantly higher vertical force than the curved trajectories (*IC*, *IL*). Specifically, the median peak vertical force in *ST* increases by 68% ($p < 0.01$) compared to static handle *SH*, and 54% ($p < 0.01$), and 24% ($p < 0.01$) compared to the curved trajectories *IC* and *IL* in standing up. In sitting down, the increases were of 48% ($p < 0.01$) compared to the static handle *SH*, and 40% ($p < 0.01$) and 25% ($p < 0.01$) compared to *IC* and *IL*, respectively. The peak horizontal handle force tended to be lower for moving handles versus the static handle (*SH*) condition, with *IC* decreasing 52% ($p<0.01$), *IL* 61% ($p<0.01$) and *SS* 52% ($p<0.01$) in standing up, and *IL* 35% ($p<0.05$) in sitting down.

**Fig. 11:**
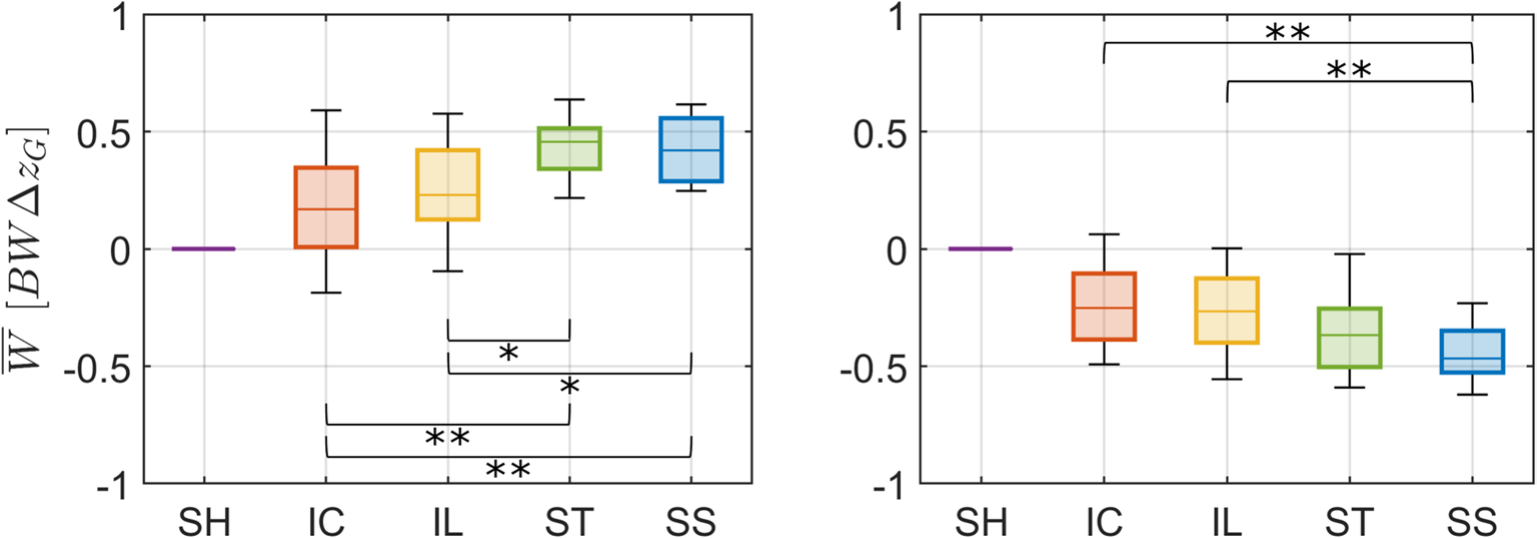
Mechanical work provided by different trajectories. Box plots (median and interquartiles, 25 and 75%) of the evaluation metric normalized work exerted by the handles $\overline{W}$ (Eq. 10) for standing up (*left*) and sitting down (*right*) supported by static handles (*SH*) and by the four types of assistance trajectories (*IC*, *IL*, *ST*, *SS*). A positive mechanical work means energy is transferred to the body, contributing to gravitational potential energy increase during standing up. A negative mechanical work means energy is dissipated, contributing to the controlled reduction of gravitational potential energy during sitting down. Statistically significant differences are indicated by * for $p < 0.05$ or by ** for $p < 0.01$

A clear advantage of moving-handle assistance is the possibility of providing (standing up) or dissipating (sitting down) part of the potential gravitational energy difference. Indeed, the assistance trajectories provided or dissipated up to about 45% of the potential energy difference, with straighter trajectories being more effective than the curved ones (Fig. 11). During standing up, the straighter trajectory *ST* provided 170% ($p<0.01$) and *SS* 149% ($p<0.01$) more energy compared with curved trajectory *IC*; and 98% ($p<0.05$) and 83% ($p<0.05$), respectively, compared with curved trajectory *IL*. During sitting down, straighter trajectory *SS* dissipated 85% ($p<0.01$) more energy than curved trajectory *IC* and 76% ($p<0.01$) more energy than *IL*.

**Fig. 12:**
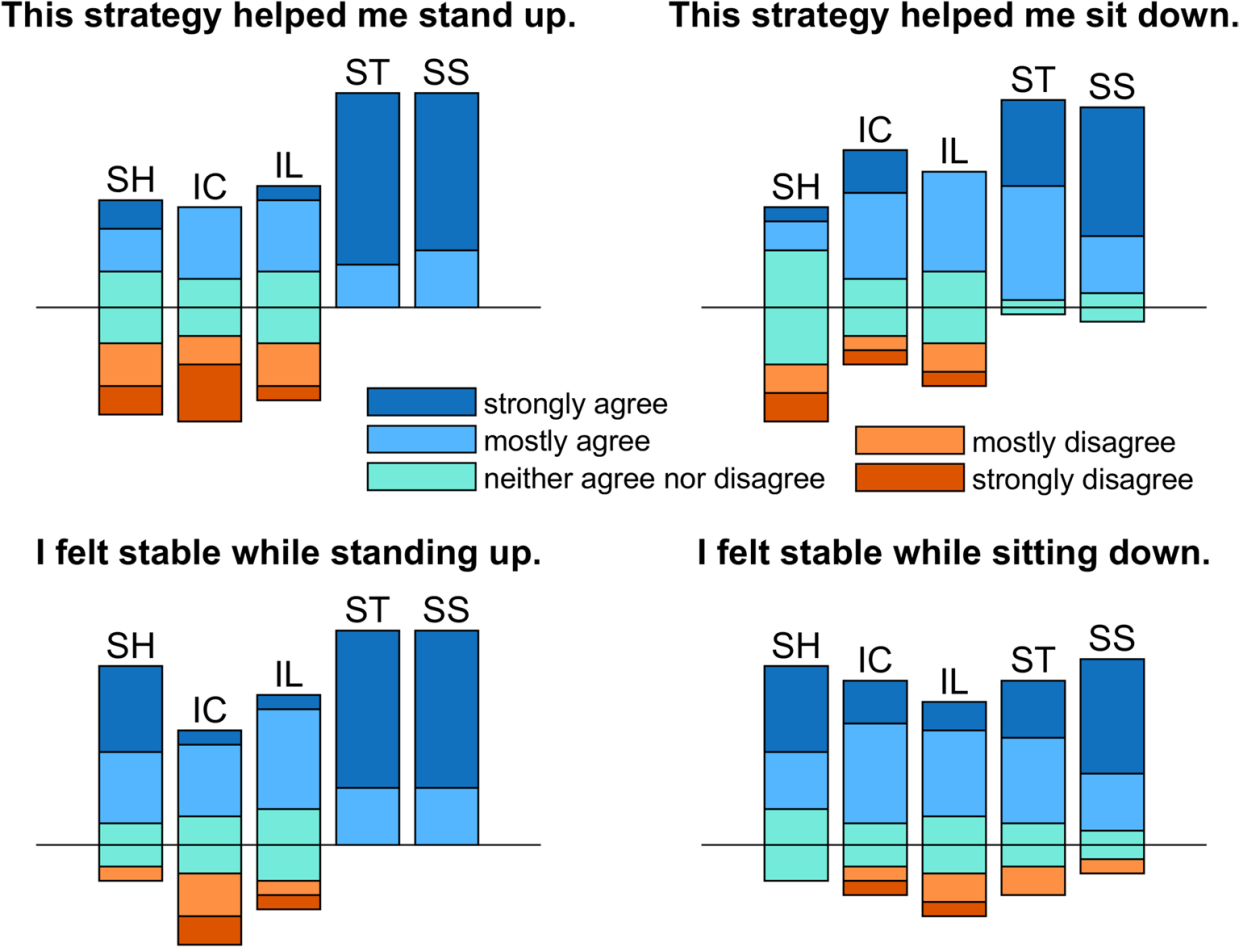
Subjective evaluation responses on perceived support and stability for STS assisted with fixed handles (*SH*), and with four assistance trajectories (*IC*, *IL*, *ST*, and *SS*), during standing up (*left*) and sitting down (*right*). The vertical bars represent 100% of the responses (15 participants) and are placed so that positive responses are above the reference horizontal line (in *black*) and negative responses are below, with sub-bars in different colors indicating the fraction of responses in each level of the scale

The straighter trajectories *SS* and *ST* were perceived as providing more support compared with the curved trajectories *IC* and *IL* (Fig. 12), a result consistent with the objective metrics showing these trajectories elicit greater vertical support forces, provide or dissipate more mechanical work, and lead to a substantial reduction in knee and hip peak moments during both standing up and sitting down. It is noteworthy that the curved trajectories *IC* and *IL* have subjective ratings similar or even poorer than the static handle *SH*, despite the reduction they led to in peak knee and hip moments. This could be related to the large hip flexion and trunk inclination they require, a motion considered awkward by some of the participants.

**Fig. 13:**
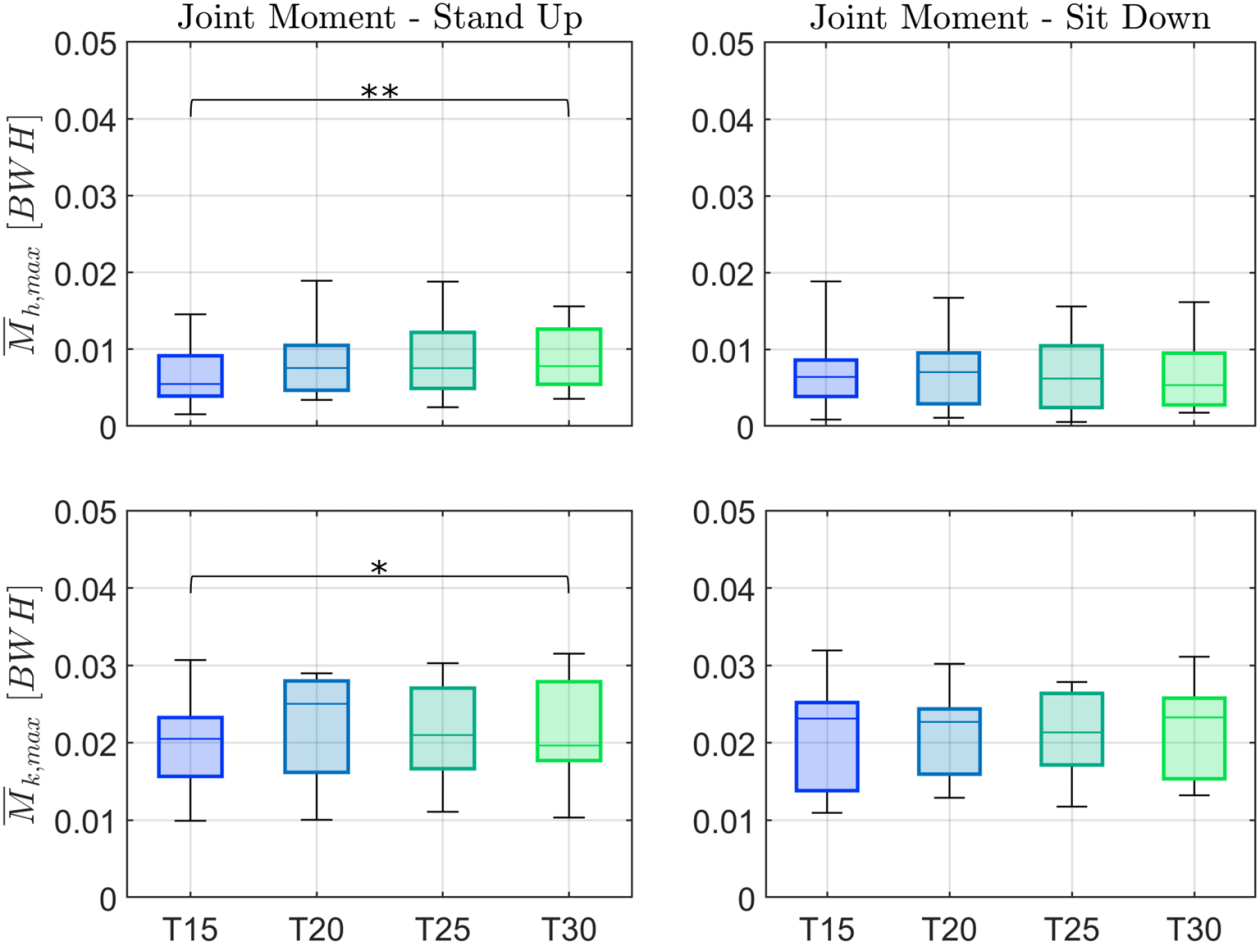
Velocity effect on leg loading. Shown are box plots (median and interquartiles, 25 and 75%) of the peak hip extension moment $\overline{M}_{h,max}$ (Eq. 6) and peak knee extension moment $\overline{M}_{k,max}$ (Eq. 7) for standing up (*left*) and sitting down (*right*). The assistance trajectory *SS* was performed at four velocities (*T15*—fastest, *T20*, *T25*, *T30*—slowest). Values were normalized by body weight and body height. Statistically significant differences are indicated by * for $p < 0.05$ or by ** for $p < 0.01$

**Fig. 14:**
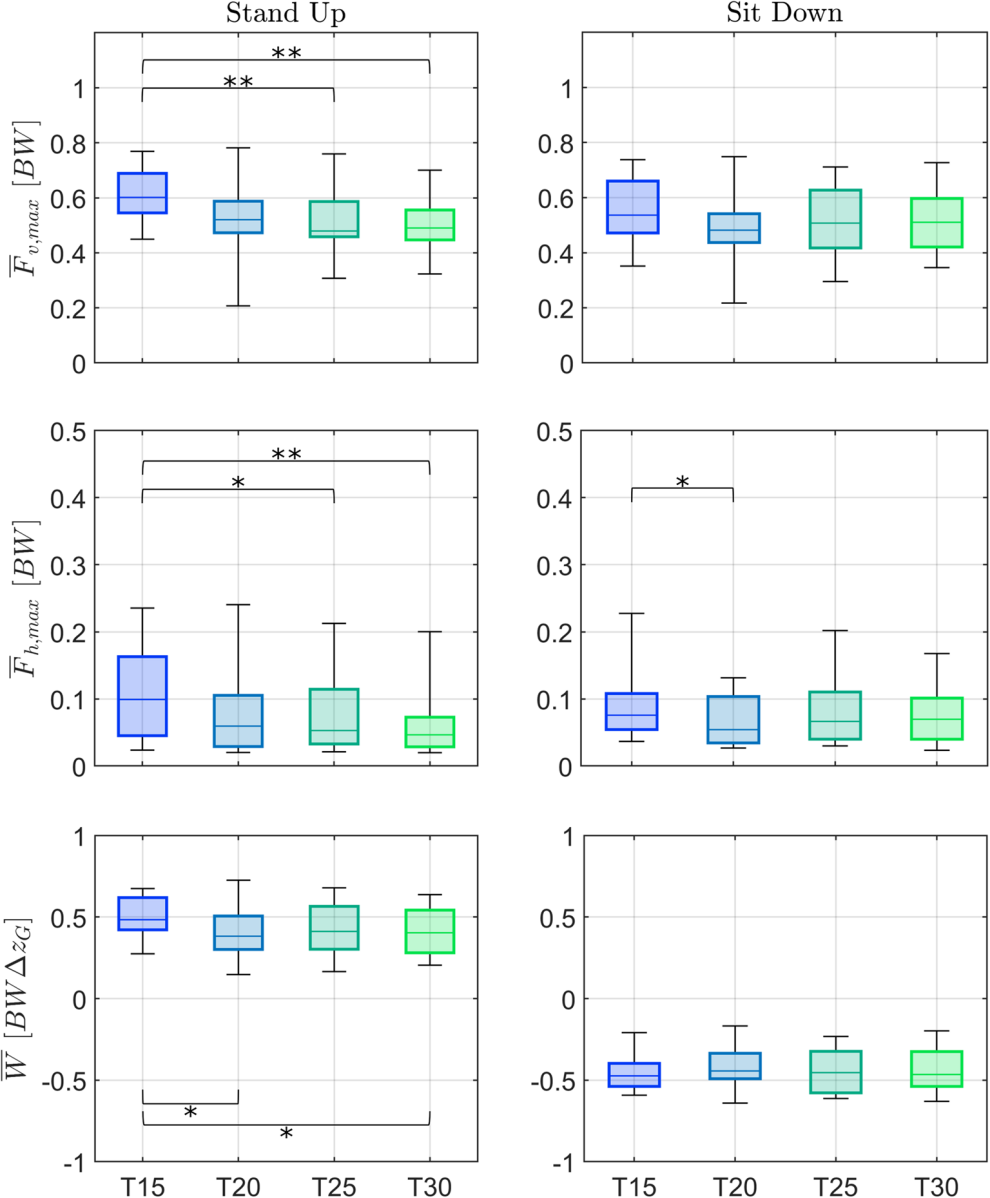
Velocity effect on handle forces. Shown are box plots (median and interquartiles, 25 and 75%) of the peak vertical force applied by the handles $\overline{F}_{v,max}$ (Eq. 8), peak horizontal force $\overline{F}_{h,max}$ (Eq. 9), and normalized mechanical work $\overline{W}$ (Eq. 10) exerted by the handles for standing up (*left*) and sitting down (*right*). The assistance trajectory *SS* was performed at four velocities (*T15*—fastest, *T20*, *T25*, *T30*—slowest). A positive mechanical work means energy is transferred to the body, contributing to gravitational potential energy increase during standing up. A negative mechanical work means energy is dissipated, contributing to the controlled reduction of gravitational potential energy during sitting down. Statistically significant differences are indicated by * for $p < 0.05$ or by ** for $p < 0.01$

**Fig. 15:**
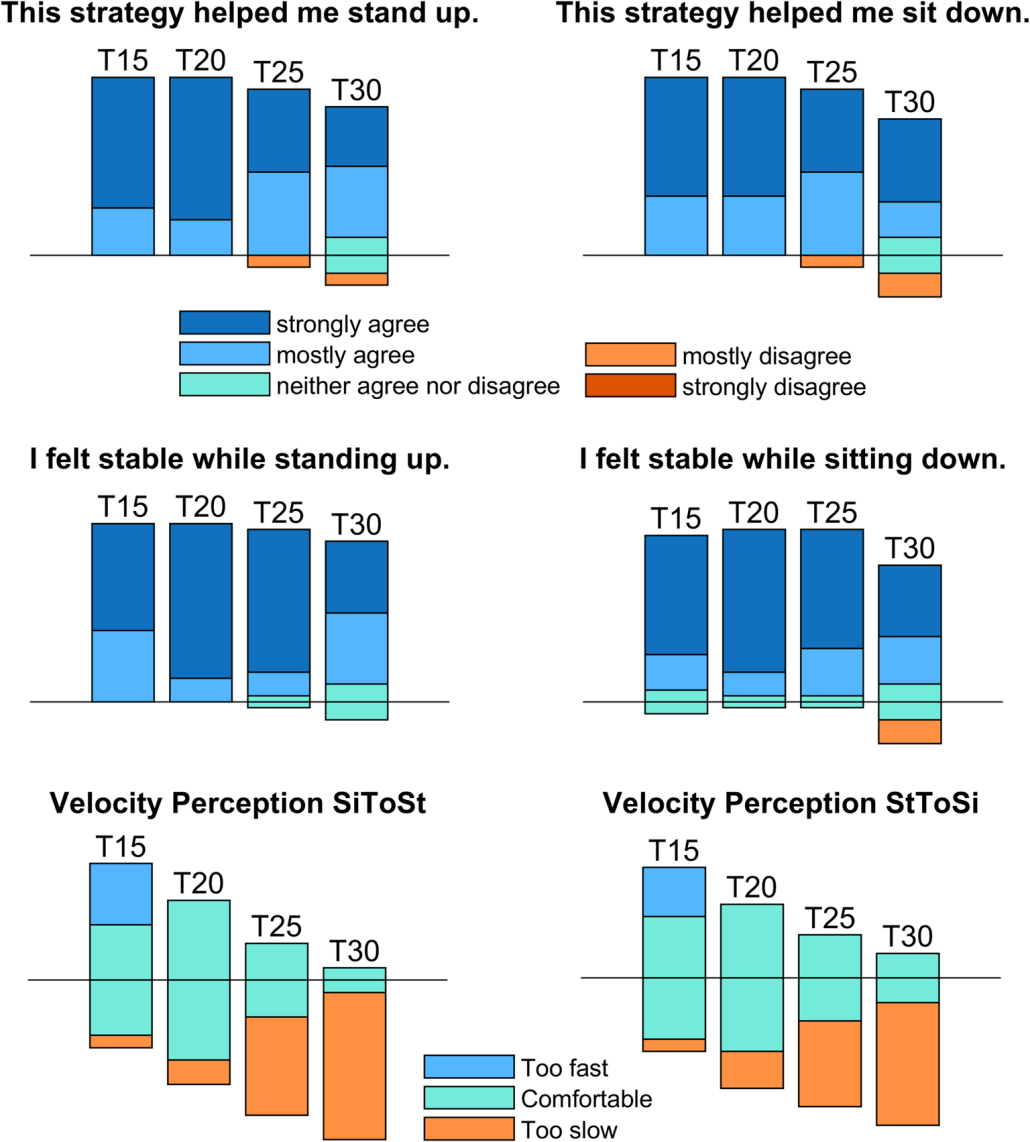
Velocity effect on subjective perception of support, stability and speed. Subjective evaluation scores by the 15 participants for *SS* at the four investigated velocity conditions, *T15* (fastest), *T20*, *T25*, and *T30* (slowest), for standing up (*left*) and sitting down (*right*). The vertical bars represent 100% of the responses (15) and are placed so that positive responses are above the reference horizontal line (in *black*) and negative responses are below, with sub-bars in different colors indicating the fraction of responses in each level of the scale

The last aim was to evaluate the effect of handle speed for the *SS* trajectory. Leg effort, handle loading, and perceived assistance were all affected by handle speed during standing up (see Figs. 13 and 14, and Tables 8 and 9 in Appendix D). The differences, however, were either marginal or limited to the comparison between the fastest and slowest velocity: during standing up the fastest condition *T15* elicited a lower peak hip moment (29%, $p<0.01$), a higher vertical handle force (23%, $p<0.01$), a higher horizontal handle force (115%, $p<0.01$), and a larger exerted mechanical handle work (20%, $p<0.05$) compared with the slowest velocity *T30*. During sitting down, only the peak horizontal handle force was affected. There was little difference in the subjectively perceived support (Fig. 15, top and middle) between the different velocity conditions, although the slowest velocity *T30* started to show a subtle decline in perceived support. This is reflected in the perception of the velocities (Fig. 15, bottom), showing that most participants considered *T30* too slow. This degradation in ratings could occur due to the sustained application of the upper extremity forces for longer periods as the duration increases, which is not taken into account in the proposed evaluation metrics based on peak lower limb joint moments.

## Discussion

Aligned with the first aim of this study, we created handle trajectories to support standing up and sitting down that can be easily implemented in robotic assistance devices such as robotic rollators. These trajectories are represented by normalized horizontal and vertical velocity profiles described by a Gaussian-like function (Eq. 1) with parameters documented in Tables 1-4. The fact that the trajectories are based on measured motion during unassisted STS and can be scaled to the user’s anthropometry and the desired velocity according to Eq. 5 ensures that the resulting kinematic patterns are within physiological ranges. Given the scarcity of information on trajectories for robotic STS assistance, we expect that the presented trajectory features and their thorough evaluation will provide a valuable reference for future studies and implementation.

It is noteworthy that the proposed handle trajectories are approximately mirrored in terms of the velocity profiles and geometrically similar between standing up and sitting down (Figs. 2 and 3), which reflects similarities in the shoulder trajectory between standing and sitting. This can be contemplated by analyzing the coefficients of the trajectories (Tables 1-4), considering that the coefficients *a* and *c* define the form of the bell-shaped components in Eq. 1, the sign of *a* indicates motion direction, and the *b* coefficients describe the “shift” in time of the bell-shaped components. Note that the magnitudes of coefficients *a* and *c* are similar for standing up and sitting down, indicating the velocity profiles are similar in magnitude. In addition to this, the similar relative shifting of the vertical components with respect to the horizontal components, with similar magnitudes and opposite signs for the differences between the *b* coefficients, indicate similar path geometries for standing up and sitting down.

For the implementation in a real robotic rollator, velocity and position profiles of the handles for a certain trajectory type (defined by coefficients *a*, *b*, and *c* in Tables 1-4), would be customized based on the desired duration (*T*), and initial ($h_{st}$) and final ($h_{si}$) handle heights as in Eq. 5. The duration *T* would be chosen based on the user’s preference and disability, with frail older adults likely selecting slower motion (larger *T*) than younger, healthier adults. Indeed, in a previous study [42], we found that the shape of the velocity profiles are similar between younger and older adults, with the difference that the overall velocity magnitude is lower in older adults, i.e. the shape of the shoulder trajectory is similar, but older adults travel the path slower, which can be represented by a larger *T*. The final and initial vertical heights would be selected based on anthropometrics (wrist heights while sitting and standing), as well as environmental constraints. Once these parameters are selected, the position and velocity trajectories in the vertical and horizontal directions for standing up and sitting down can be computed offline in the rollator’s embedded computer using the previously stored trajectory coefficients (Tables 1-4), and stored, for instance, as lookup tables, which would feed the closed-loop position/velocity controllers of the rotational or linear actuators, depending on the mechanical realization. The beginning of the handle motion could be indicated by sound or tactile (vibration) clues. Time delays would depend on system architecture and components but would typically not exceed some tens of milliseconds which is not critical in this application.

While the proposed physiological trajectories were shown to substantially reduce lower limb load during STS, they represent only a subset of all possible trajectories. Other trajectories, with different geometric shapes and velocity profiles, could potentially lead to even better support. A systematic search for optimal trajectories that reduce not only lower but also upper extremity loads and ensure user and rollator stability could be the focus of future computational or experimental studies. For instance, a predictive simulation framework, such as in [38] or [35], could be formulated to search for optimal parameters of the velocity profiles in Eq. 1 by using a validated model of the human-robot system and an appropriate cost function as performance criterion. The current results cold help to inform these predictive simulations. Experimentally, a human–in–the–loop approach such as in [43] could be implemented, with each standing up or sitting down by the participant providing a “function evaluation” of the cost function in the optimization. This study provides an important first step, showing that particularly the straighter *ST* and *SS* trajectories led to substantial improvements in leg effort and perceived assistance compared with a static rollator handle.

The second aim was to evaluate the effect of the different trajectories on leg loading, handle support and perceived assistance. Primarily, a successful support trajectory would reduce the burden on the lower extremity. Particularly older frail adults would benefit from lower required leg moments as general capacity declines with age [6]. Gross et al. [7], for instance, reports reductions of 35% and 55% in maximal hip and knee extension moments in older adults, respectively, directly affecting the ability to stand up and sit down. We show that static handles can reduce the peak hip moments (by about 50%, in line with the reductions provided by armrests reported in the literature [44]), but do not reduce knee moments. Moving handles, particularly straighter trajectories, further reduce the required peak hip moment to nearly zero, while reducing knee extension moments by over 50% compared to static handles. Therefore, moving handles appear to favor a more uniform reduction in knee and hip moments in contrast to static handles. These findings confirm our first hypothesis that providing moving-handle assistance reduces the lower limb loading during standing up and sitting down compared to static handles assistance. However, we have also shown that the extent of the benefit is trajectory-dependent, with the trajectories *ST* and *SS* providing larger gains than *IC* and *IL*. This refutes our second hypothesis that handle trajectories closer to the reference shoulder trajectories for unassisted STS, which correspond to the curved shapes *IC* and *IL*, would lead to better performance compared to their straighter counterparts *ST* and *SS*.

This promising leg effort reduction comes at the expense of larger vertical handle forces (up to 60% compared with 40% of body weight by static handles) and thus load on the arms/upper body. A high prevalence of shoulder pain and injury among wheelchair users has long been associated with the large and repetitive upper extremity loads in activities such as wheelchair locomotion, weight-relief lifting, and transfers [45]. The weight-relief lifting, which involves a maneuver to reduce pressure on buttocks and hips to prevent pressure sores, can be compared to rollator assisted standing up and sitting down and was associated with particularly large upper extremity joint moments [46] and glenohumeral contact forces [47]. These results indicate there is a trade-off between unloading the legs and overloading the arms. This is likely to play an important role in older adults, as skeletal muscle loss due to aging affects both lower and upper extremities [48]. Rollators providing forearm or trunk support can potentially mitigate this by reducing the load transferred through the wrist, elbow and shoulder joints. In terms of control strategies, impedance control or real-time adaptive trajectories could not only potentially reduce excessive upper-limb loads, but also further personalize assistance and enforce stability. Therefore, the next research steps include examining the handle trajectory effects directly with older and frail adults and investigating more adaptive control strategies.

The increase in vertical handle force for moving-handle trajectories is generally associated with a decrease in horizontal forces, with reductions of over 50% observed in peak values. Interestingly, most participants changed from pulling static handles to pushing moving handles during both standing up and sitting down. This clearly indicates a change in the nature of the interaction with the assistive device. As evidenced in Fig. 4, the COM position is posterior to the handles and the feet with the static handles, a configuration that requires pulling the handle during slow standing up and sitting down, while the predominantly horizontal pose of the arm facilitates the transmission of horizontal rather than vertical forces. In contrast, during all moving-handle trajectories the arms are stretched vertically and the COM remains above the handles, either vertically aligned (*IC* and *IL*) or in a slightly anterior position (*ST* and *SS*), which favors the transmission of vertical forces.

It is expected that this reduction in peak horizontal force increases the stability of the rollator during the STS transfers. Large horizontal forces exerted on current passive, lightweight rollators lead to a greater risk of the rollator tipping over. Our experimental setup does not accurately represent this because the Robotic Assistance Simulator device (Fig. 1) is heavy and can virtually transmit any level of horizontal force without tipping over or slipping. For this reason, the observed horizontal forces in *SH* are likely larger than those applied in real rollators. However, the results for the static condition can still be considered a valid reference for comparison, as the reduction in leg moments using static handles are likely an overestimation of the reduction achieved by real rollators. Nevertheless, it is important to emphasize that the current study does not directly evaluate stability or the risk of tipping over during the practical use of a real robotic rollator. Given the severity of injuries associated with falls involving rollators [17], future studies should address these important factors in evaluating conventional as well as robotic rollators with STS assistance.

Moving handles provided a substantial fraction of the potential energy required to move the body from a lower to a higher position (over 40% for the straighter trajectories) during standing up. During sitting down, on the contrary, the moving handles dissipate the surplus energy and help control the motion when the body moves down (over 35% for the straighter trajectories). Although this study did not investigate how this energy is redistributed among body segments as it is transferred to or from the body thorough the handles, the possibility of providing or dissipating mechanical work is certainly an advantage that is absent in static handles, for which all the required energy difference needs to be provided or dissipated by the muscles.

Participants showed preference for the straighter trajectories *SS* and *ST* over the static handle and curved trajectories *IC* and *IL*, which further refutes our second hypothesis. This is interesting, as the motion profiles of the curved trajectories are closer to the unassisted biological shoulder profiles. This can be explained by the superior performance of the straighter trajectories in terms of unloading the leg and providing or dissipating mechanical work, indicating that these effects positively influence the perceived handle assistance. The curved trajectories are also associated with large trunk inclinations and hip flexion angles (Fig. 6), which were explicitly referred to by some of the participants using words as “uncomfortable”, “unnatural”, “weird”, “not intuitive”, and “unstable” (see collection of participant comments on the different trajectories in Appendix E). Prominent trunk forward inclination at seat off or seat on is a common strategy to guarantee stability and transfer momentum [2]. With moving handles, motion is slower, meaning smaller inertial effects, and handles remain approximately under the participant’s COM, ensuring static equilibrium over nearly all the standing up and sitting down activity. Thus, in the actively assisted STS, trunk inclination proves unnecessary and even inconsistent with the slower dynamics. Note that the subjective evaluation does not use validated questionnaires, as we wanted to evaluate the isolated effect of the support (versus the robot) during the specific movements.

The third aim was to evaluate how handle speed affects leg loading, handle forces and perceived support. The quasi-static nature of the assisted STS described above is corroborated by the marginal differences among the slower conditions *T20*, *T25*, and *T30* in most metrics. However, we did find that inertial effects start playing a role in the fastest velocity *T15*. Such an effect of movement speed is also found in unassisted STS [49], with small contribution of inertial effects to the knee and hip peak moments during movements taking 2.5 s or more, so that further increases in duration cause only small changes in joint moments. Considering that this study’s reported duration includes the time between the start of trunk motion and *seat off*, we estimate that this critical duration corresponds approximately to the condition *T20*. This is indeed where our effects seem to plateau. A duration of $T=1$ s is closer to the average duration of unassisted STS and would be characterized by much larger inertial effects. However, velocities greater than the one for $T=1.5$ s (*T15*) were considered too fast for assisted STS in pilot tests during the protocol design phase, a finding later corroborated by the subjective perception of the participants on velocity (Fig. 15), with many of the young healthy participants evaluating *T15* as too fast. This indicates that assisted and unassisted STS are different in nature.

The generally small influence of the velocity within the investigated range refutes our third hypothesis that handle speed would have a substantial effect on the outcomes, and indicates that trajectory shape rather than velocity determines the participant’s biomechanical response. This means that the handle velocity can be selected according to the user’s preferences without significantly affecting peak leg loads and transferred energy. However, it is interesting to observe that participants felt more comfortable at the speed *T20*, over faster or slower handle movements. This is possibly because longer durations imply the same loads are applied over longer periods of time, unnecessarily prolonging the effort and likely increasing fatigue and energy consumption over time. While faster movements could be destabilizing. This time-dependency is not taken into account in the leg loading outcomes we used and should be considered in future studies. The effect of handle velocity on safety and perceived assistance, among other outcomes, remains to be investigated.

While providing important insights on the human response to moving-handle assistance, the group of healthy young participants in this study does not fully represent the range of potential users of this assistive technology. Further experimental assessments with the target population, i.e. individuals with difficulties standing up such as older frail adults, is required and is the focus of ongoing research. In fact, preliminary results of an ongoing study with frail older adults using similar experimental design and setup reveal that frail older adults tend to apply lower normalized vertical forces to the handles compared to younger healthy adults. This potential difference in response emphasizes the need for further experimental investigations.

Another limitation of this study is gender imbalance, as only 3 of the participants were female. It has been shown, for instance, that there are gender differences in joint angles, with women presenting more knee and ankle flexion in mid-phases and less hip flexion in later phases compared to men in unassisted standing up [50]. An inspection of the differences between women and men responses in our participant group for standing up shows that the average of the evaluation metrics for the 3 female participants are mainly within 15% of the ones for the 12 male participants, except for lower normalized horizontal handle forces, lower normalized work for the curved trajectories, and larger normalized hip moments for the straighter trajectories. Furthermore, other factors such as body weight have not been considered. As different STS kinematics and kinetics have been observed in obese individuals, e.g. [51], future research should evaluate if body weight affects biomechanical loading and support perception.

## Conclusion

We present simple, parameterized support trajectories for robotic rollators equipped with moving handles to assist standing up or sitting down movements. These biological trajectories are based on unassisted shoulder patterns and can be scaled to the user’s anthropometry and desired velocity. The proposed moving handle trajectories substantially reduce leg loading (peak hip and knee extension moments), increase the vertical handle support, and improve the subjective assistance perception compared with static handles (representing a conventional passive rollator) in young adults, while providing or dissipating a large fraction of the potential energy difference in standing and sitting, respectively. These positive effects are larger for the straighter trajectories compared with the more curved trajectories, with reductions of joint moments exceeding 70% for the hip and 50% for the knee in comparison to the static handle for both standing up and sitting down. The moving handles caused a shift in user-rollator interaction from large horizontal pulling forces with static handles to lower horizontal forces on moving handles (potentially reducing rollator tipping risk) but with higher vertical forces (with a potentially excessive upper body demand in older adults). These results evidence the potential of the proposed moving handle assistive trajectories to reduce lower extremity mechanical demand and improve the quality of life of rollator users with difficulties standing up. This study is the first thorough experimental investigation of human-robot interaction (HRI) in the case of actuated handle support. However, it must be emphasized that the participant group is composed of young, healthy adults and that the baseline condition does not fully represent the STS with passive rollators. In our ongoing research, building on top of the present paper, we are validating these results in individuals with difficulties standing up, including frail older adults, and comparing them to actual passive rollators.

## Additional file

Additional file 1: Hip Extension Moment for Different Assistance Trajectories in Standing; Description: peak hip extension moment during standing up normalized by body weight and participant’s height for the different assistance trajectories (columns: *SH*, *IC*, *IL*, *ST*, *SS*) and all 15 participants (rows) (Eq. 6, $\overline{M}_{h,max}$, Table 6).

Additional file 2: Knee Extension Moment for Different Assistance Trajectories in Standing; Description: peak knee extension moment during standing up normalized by body weight and participant’s height for the different assistance trajectories (columns: *SH*, *IC*, *IL*, *ST*, *SS*) and all 15 participants (rows) (Eq. 7, $\overline{M}_{k,max}$, Table 6).

Additional file 3: Vertical Handle Force for Different Assistance Trajectories in Standing; Description: peak vertical force applied by both handles during standing up normalized by body weight for the different assistance trajectories (columns: *SH*, *IC*, *IL*, *ST*, *SS*) and all 15 participants (rows) (Eq. 8, $\overline{F}_{v,max}$, Table 6).

Additional file 4: Horizontal Handle Force for Different Assistance Trajectories in Standing; Description: peak absolute horizontal force applied by both handles during standing up normalized by body weight for the different assistance trajectories (columns: *SH*, *IC*, *IL*, *ST*, *SS*) and all 15 participants (rows) (Eq. 9, $\overline{F}_{h,max}$, Table 6).

Additional file 5: Mechanical Work by the Handles for Different Assistance Trajectories in Standing; Description: mechanical work exerted by both handles during sitting down normalized by the difference in potential gravitational energy difference between standing and sitting for the different assistance trajectories (columns: *SH*, *IC*, *IL*, *ST*, *SS*) and all 15 participants (rows) (Eq. 10, $\overline{W}$, Table 6).

Additional file 6: Hip Extension Moment for Different Assistance Trajectories in Sitting; Description: peak hip extension moment during sitting down normalized by body weight and participant’s height for the different assistance trajectories (columns: *SH*, *IC*, *IL*, *ST*, *SS*) and all 15 participants (rows) (Eq. 6, $\overline{M}_{h,max}$, Table 7).

Additional file 7: Knee Extension Moment for Different Assistance Trajectories in Sitting; Description: peak knee extension moment during sitting down normalized by body weight and participant’s height for the different assistance trajectories (columns: *SH*, *IC*, *IL*, *ST*, *SS*) and all 15 participants (rows) (Eq. 7, $\overline{M}_{k,max}$, Table 7).

Additional file 8: Vertical Handle Force for Different Assistance Trajectories in Sitting; Description: peak vertical force applied by both handles during sitting down normalized by body weight for the different assistance trajectories (columns: *SH*, *IC*, *IL*, *ST*, *SS*) and all 15 participants (rows) (Eq. 8, $\overline{F}_{v,max}$, Table 7).

Additional file 9: Horizontal Handle Force for Different Assistance Trajectories in Sitting; Description: peak absolute horizontal force applied by both handles during sitting down normalized by body weight for the different assistance trajectories (Eq. 9, $\overline{F}_{h,max}$, Table 7).

Additional file 10: Mechanical Work by the Handles for Different Assistance Trajectories in Sitting; Description: mechanical work exerted by both handles during sitting down normalized by the difference in potential gravitational energy difference between standing and sitting for the different assistance trajectories (columns: *SH*, *IC*, *IL*, *ST*, *SS*) and all 15 participants (rows) (Eq. 10, $\overline{W}$, Table 7).

Additional file 11: Hip Extension Moment for Different Velocities in Standing; Description: peak hip extension moment during standing up normalized by body weight and participant’s height for the different velocities (columns: *T15*, *T20*, *T25*, *T30*) and all 15 participants (rows) (Eq. 6, $\overline{M}_{h,max}$, Table 8).

Additional file 12: Knee Extension Moment for Different Velocities in Standing; Description: peak knee extension moment during standing up normalized by body weight and participant’s height for the different velocities (columns: *T15*, *T20*, *T25*, *T30*) and all 15 participants (rows) (Eq. 7, $\overline{M}_{k,max}$, Table 8).

Additional file 13: Vertical Handle Force for Different Velocities in Standing; Description: peak vertical force applied by both handles during standing up normalized by body weight for the different velocities (columns: *T15*, *T20*, *T25*, *T30*) and all 15 participants (rows) (Eq. 8, $\overline{F}_{v,max}$, Table 8).

Additional file 14: Horizontal Handle Force for Different Velocities in Standing; Description: peak absolute horizontal force applied by both handles during standing up normalized by body weight for the different velocities (columns: *T15*, *T20*, *T25*, *T30*) and all 15 participants (rows) (Eq. 9, $\overline{F}_{h,max}$, Table 8).

Additional file 15: Mechanical Work by the Handles for Different Velocities in Standing; Description: mechanical work exerted by both handles during sitting down normalized by the difference in potential gravitational energy difference between standing and sitting for the different velocities (columns: *T15*, *T20*, *T25*, *T30*) and all 15 participants (rows) (Eq. 10, $\overline{W}$, Table 8).

Additional file 16: Hip Extension Moment for Different Velocities in Sitting; Description: peak hip extension moment during sitting down normalized by body weight and participant’s height for the different velocities (columns: *T15*, *T20*, *T25*, *T30*) and all 15 participants (rows) (Eq. 6, $\overline{M}_{h,max}$, Table 9).

Additional file 17: Knee Extension Moment for Different Velocities in Sitting; Description: peak knee extension moment during sitting down normalized by body weight and participant’s height for the different velocities (columns: *T15*, *T20*, *T25*, *T30*) and all 15 participants (rows) (Eq. 7, $\overline{M}_{k,max}$, Table 9).

Additional file 18: Vertical Handle Force for Different Velocities in Sitting; Description: peak vertical force applied by both handles during sitting down normalized by body weight for the different velocities (columns: *T15*, *T20*, *T25*, *T30*) and all 15 participants (rows) (Eq. 8, $\overline{F}_{v,max}$, Table 9).

Additional file 19: Horizontal Handle Force for Different Velocities in Sitting; Description: peak absolute horizontal force applied by both handles during sitting down normalized by body weight for the different velocities (columns: *T15*, *T20*, *T25*, *T30*) and all 15 participan ts (rows) (Eq. 9, $\overline{F}_{h,max}$, Table 9).

Additional file 20: Mechanical Work by the Handles for Different Velocities in Sitting; Description: mechanical work exerted by both handles during sitting down normalized by the difference in potential gravitational energy difference between standing and sitting for the different velocities (columns: *T15*, *T20*, *T25*, *T30*) and all 15 participants (rows) (Eq. 10, $\overline{W}$, Table 9).

## Figures and Tables

**Fig. 16:**
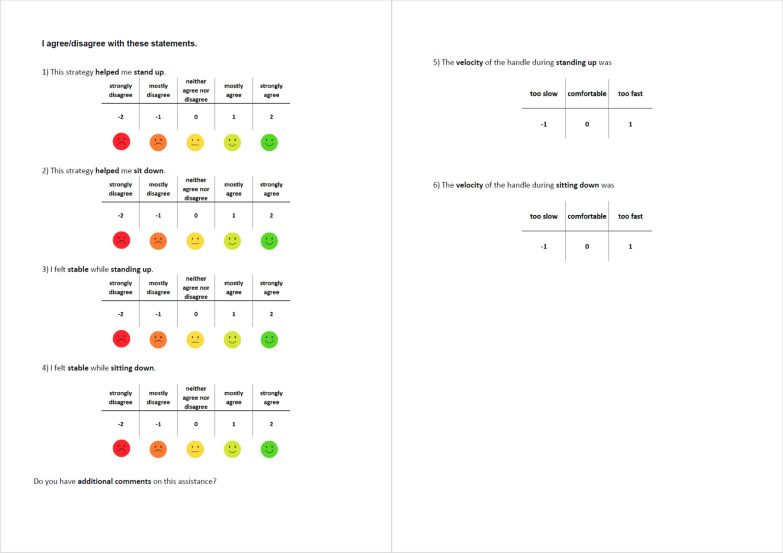
Questionnaire used for subjective evaluation of each assistance condition by the participants as explained in “Experimental protocol” section

## Data Availability

All the coefficients required to reproduce the proposed trajectories are provided in the Appendix of the manuscript. All the descriptive statistics are provided in Tables in the Appendix. All data on computed metrics for all participants and conditions used for the statistical analyses are provided within the Supplementary material in the form of spreadsheets. The original experimental data on measured kinematics, handle forces and ground reaction forces can be provided by the corresponding author upon reasonable request.

## Appendix A: Coefficients of proposed trajectories

The coefficients of Eq. 1 for the four proposed trajectories are provided in Tables 1 (horizontal velocity for standing up), 2 (vertical velocity for standing up), 3 (horizontal velocity for sitting down), and 4 (vertical velocity for sitting down).

**Table 1:** Coefficients of parameterized horizontal velocity patterns for standing up as in Eq. 1

	$a_{1,u,h}$	$b_{1,u,h}$	$c_{1,u,h}$	$a_{2,u,h}$	$b_{2,u,h}$	$c_{2,u,h}$
IC	1.9291	$-$0.1945	0.3857	$-$0.3587	0.5373	0.5048
IL	1.8326	$-$0.2107	0.3072	–	–	–
ST	1.9180	0.0761	0.2936	–	–	–
SS	0.9590	0.0761	0.5871	–	–	–

**Table 2:** Coefficients of parameterized vertical velocity patterns for standing up as in Eq. 1

	$a_{1,u,v}$	$b_{1,u,v}$	$c_{1,u,v}$	$a_{2,u,v}$	$b_{2,u,v}$	$c_{2,u,v}$
IC	1.6872	0.3197	0.3741	$-$0.7313	$-$0.1551	0.2438
IL	1.6180	0.3629	0.2799	–	–	–
ST	1.5426	0.0761	0.2936	–	–	–
SS	1.5426	0.0761	0.2936	–	–	–

**Table 3:** Coefficients of parameterized horizontal velocity patterns for sitting down as in Eq. 1

	$a_{1,d,h}$	$b_{1,d,h}$	$c_{1,d,h}$	$a_{2,d,h}$	$b_{2,d,h}$	$c_{2,d,h}$
IC	$-$1.8316	1.1888	0.4322	0.5163	0.5168	0.4428
IL	$-$1.7441	1.2205	0.3228	–	–	–
ST	$-$1.8707	0.9280	0.3010	–	–	–
SS	$-$0.9353	0.9280	0.6020	–	–	–

**Table 4:** Coefficients of parameterized vertical velocity patterns for sitting down as in Eq. 1

	$a_{1,d,v}$	$b_{1,d,v}$	$c_{1,d,v}$	$a_{2,d,v}$	$b_{2,d,v}$	$c_{2,d,v}$
IC	$-$1.6458	0.6530	0.3425	0.6868	1.1735	0.1613
IL	$-$1.6223	0.6355	0.2791	–	–	–
ST	$-$1.5046	0.9280	0.3010	–	–	–
SS	$-$1.5046	0.9280	0.3010	–	–	–

## Appendix B: Participants’ information

To assess our support profiles, we recruited 15 young able-bodied adults, with individual gender, age, mass, and height reported in Table 5 (3 female; $27.5 \pm 4.9$ yrs.; $69.2 \pm 10.0$ kg; $1.74 \pm 0.05$ m).

**Table 5:** Gender, age, mass and height of the participants

Participant	Gender	Age (years)	Mass (kg)	Height (m)
P01	m	24	73.1	1.805
P02	m	24	67.1	1.740
P03	f	27	50.1	1.645
P04	m	24	82.9	1.815
P05	m	39	72.2	1.735
P06	m	33	62.6	1.720
P07	m	34	58.5	1.695
P08	m	25	62.2	1.830
P09	m	24	68.1	1.730
P10	m	32	74.5	1.745
P11	m	20	69.9	1.790
P12	m	30	81.9	1.745
P13	f	26	63.0	1.720
P14	f	26	62.0	1.695
P15	m	25	89.8	1.760

## Appendix C: Questionnaire: subjective evaluation

After each trial of blocks 3 and 4 of the experimental protocol (“Experimental protocol” section), the participants were asked to provide a subjective evaluation on the level of perceived support and stability on a 5-point scale, as well as on the perceived velocity on a 3-point scale, for standing up and sitting down, except for the condition *SH*, for which the evaluation of perceived velocity does not apply. Refer to the questionnaire presented to the participants in Fig. 16.

## Appendix D: Descriptive statistics of evaluation metrics

The different assistance trajectories (*IC*, *IL*, *ST*, *SS*) and the support by static handles (*SH*) were compared in terms of the 5 evaluation metrics introduced in “Data evaluation” section: maximal normalized hip extension moment (Eq. 6, $\overline{M}_{h,max}$), maximal normalized knee extension moment (Eq. 7, $\overline{M}_{k,max}$), maximal normalized vertical force applied by the handles (Eq. 8, $\overline{F}_{v,max}$), maximal absolute value of the normalized horizontal force applied by the handles (Eq. 9, $\overline{F}_{h,max}$), and normalized work exerted by the handles (Eq. 10, $\overline{W}$). The descriptive statistics median and Interquartile Range (25% - 75%) are reported in Table 6 for standing up and in Table 7 for sitting down.

**Table 6:** Evaluation metrics for standing up supported by static handles (*SH*) and by the four types of assistance trajectories (*IC*, *IL*, *ST*, *SS*), where $\overline{M}_{h,max}$ is the maximal normalized hip extension moment (Eq. 6), $\overline{M}_{k,max}$ is the maximal normalized knee extension moment (Eq. 7), $\overline{F}_{v,max}$ is the maximal vertical force applied by the handles (Eq. 8), $\overline{F}_{h,max}$ is the maximal absolute value of the horizontal force applied by the handles (Eq. 9), and $\overline{W}$ is the normalized work exerted by the handles (Eq. 10). Median (50%) and IQR (25–75%) values are reported. The last column lists the pairs of conditions with statistically significant differences, with * for $p < 0.05$ or ** for $p < 0.01$

Stand Up	%	SH	IC	IL	ST	SS	$p < 0.05$ (*); $p < 0.01$ (**)
$\overline{M}_{h,max}$ [*BWH*]	50	0.0212	0.0180	0.0091	0.0056	0.0059	SH-IL**, SH-ST**, SH-SS**,
	25	0.0133	0.0055	0.0051	0.0031	0.0045	IC-ST*
	75	0.0303	0.0214	0.0172	0.0090	0.0146	
$\overline{M}_{k,max}$ [*BWH*]	50	0.0450	0.0378	0.0314	0.0190	0.0220	SH-IL*, SH-ST**, SH-SS**,
	25	0.0392	0.0325	0.0271	0.0127	0.0149	IC-ST**, IC-SS**, IL-ST**
	75	0.0515	0.0399	0.0381	0.0246	0.0309	
$\overline{F}_{v,max}$	50	0.366	0.400	0.498	0.616	0.529	SH-ST**, IC-ST**, IC-SS*,
[*BW*]	25	0.277	0.323	0.322	0.541	0.454	IL-ST**
	75	0.519	0.521	0.537	0.728	0.605	
$\overline{F}_{h,max}$	50	0.149	0.071	0.058	0.110	0.071	SH-IC**, SH-IL**, SH-SS**,
[*BW*]	25	0.105	0.050	0.043	0.046	0.038	ST-SS**
	75	0.194	0.127	0.143	0.177	0.105	
$\overline{W}$	50	N.A	0.169	0.230	0.456	0.420	IC-ST**, IC-SS**, IL-ST*,
$[BW \Delta z_G]$	25	N.A.	$-$0.002	0.118	0.322	0.267	IL-SS*
	75	N.A.	0.359	0.427	0.514	0.567

**Table 7:** Evaluation metrics for sitting down supported by static handles (*SH*) and by the four types of assistance trajectories (*IC*, *IL*, *ST*, *SS*), where $\overline{M}_{h,max}$ is the maximal normalized hip extension moment (Eq. 6), $\overline{M}_{k,max}$ is the maximal normalized knee extension moment (Eq. 7), $\overline{F}_{v,max}$ is the maximal vertical force applied by the handles (Eq. 8), $\overline{F}_{h,max}$ is the maximal absolute value of the horizontal force applied by the handles (Eq. 9), and $\overline{W}$ is the normalized work exerted by the handles (Eq. 10). Median (50%) and IQR (25–75%) values are reported. The last column lists the pairs of conditions with statistically significant differences, with * for $p < 0.05$ or ** for $p < 0.01$

Sit Down	%	SH	IC	IL	ST	SS	$p < 0.05$ (*); $p < 0.01$ (**)
$\overline{M}_{h,max}$	50	0.0231	0.0118	0.0106	0.0089	0.0054	SH-IC**, SH-IL**, SH-ST**,
[*BWH*]	25	0.0152	0.0047	0.0052	0.0060	0.0030	SH-SS**
	75	0.0327	0.0148	0.0140	0.0130	0.0095	
$\overline{M}_{k,max}$	50	0.0449	0.0341	0.0316	0.0226	0.0230	SH-ST**, SH-SS**, IC-ST**,
[*BWH*]	25	0.0406	0.0264	0.0254	0.0141	0.0158	IC-SS* , IL-ST*
	75	0.0496	0.0378	0.0347	0.0271	0.0274	
$\overline{F}_{v,max}$	50	0.408	0.435	0.486	0.607	0.558	SH-ST**, IC-ST**, IL-ST**
[*BW*]	25	0.340	0.391	0.384	0.470	0.438	
	75	0.500	0.615	0.601	0.673	0.624	
$\overline{F}_{h,max}$	50	0.120	0.067	0.078	0.079	0.068	SH-IL*
[*BW*]	25	0.082	0.035	0.038	0.058	0.035	
	75	0.152	0.097	0.091	0.131	0.112	
$\overline{W}$	50	N.A	$-$0.252	$-$0.266	$-$0.367	$-$0.467	IC-SS**, IL-SS**
$[BW \Delta z_G]$	25	N.A.	$-$0.404	$-$0.408	$-$0.509	$-$0.531	
	75	N.A.	$-$0.104	$-$0.125	$-$0.251	$-$0.332	

The different velocity conditions (*T15*, *T20*, *T25*, *T30*) were compared in terms of the same 5 evaluation metrics. The descriptive statistics median and Interquartile Range (25% - 75%) are reported in Table 8 for standing up and in Table 9 for sitting down.

**Table 8:** Evaluation metrics for standing up supported by the assistance trajectory *SS* for the velocities *T*15 ($T=1.5$ s), *T*20 ($T=2.0$ s), *T*25 ($T=2.5$ s), and *T*30 ($T=3.0$ s), where $\overline{M}_{h,max}$ is the maximal normalized hip extension moment (Eq. 6), $\overline{M}_{k,max}$ is the maximal normalized knee extension moment (Eq. 7), $\overline{F}_{v,max}$ is the maximal vertical force applied by the handles (Eq. 8), $\overline{F}_{h,max}$ is the maximal absolute value of the horizontal force applied by the handles (Eq. 9), and $\overline{W}$ is the normalized work exerted by the handles (Eq. 10). Median (50%) and IQR (25–75%) values are reported. The last column lists the pairs of conditions with statistically significant differences, with * for $p < 0.05$ or ** for $p < 0.01$

Stand Up	%	T15	T20	T25	T30	$p < 0.05$ (*); $p < 0.01$ (**)
$\overline{M}_{h,max}$	50	0.0055	0.0075	0.0075	0.0078	V15-V30**
[*BWH*]	25	0.0035	0.0045	0.0049	0.0051	
	75	0.0097	0.0106	0.0124	0.0129	
$\overline{M}_{k,max}$	50	0.0205	0.0250	0.0210	0.0197	V15-V30*
[*BWH*]	25	0.0156	0.0156	0.0165	0.0176	
	75	0.0236	0.0283	0.0272	0.0284	
$\overline{F}_{v,max}$	50	0.602	0.520	0.479	0.490	V15-V25**, V15-V30**
[*BW*]	25	0.544	0.466	0.457	0.441	
	75	0.691	0.595	0.587	0.561	
$\overline{F}_{h,max}$	50	0.099	0.059	0.053	0.046	V15-V25*, V15-V30**
[*BW*]	25	0.043	0.028	0.031	0.028	
	75	0.171	0.112	0.121	0.074	
$\overline{W}$	50	0.484	0.382	0.412	0.404	V15-V20*, V15-V30*
$[BW \Delta z_G]$	25	0.421	0.293	0.293	0.275	
	75	0.625	0.507	0.566	0.552

**Table 9:** Evaluation metrics for sitting down supported by the assistance trajectory *SS* for the velocities *T*15 ($T=1.5$ s), *V*20 ($T=2.0$ s), *T*25 ($T=2.5$ s), and *T*30 ($T=3.0$ s), where $\overline{M}_{h,max}$ is the maximal normalized hip extension moment (Eq. 6), $\overline{M}_{k,max}$ is the maximal normalized knee extension moment (Eq. 7), $\overline{F}_{v,max}$ is the maximal vertical force applied by the handles (Eq. 8), $\overline{F}_{h,max}$ is the maximal absolute value of the horizontal force applied by the handles (Eq. 9), and $\overline{W}$ is the normalized work exerted by the handles (Eq. 10). Median (50%) and IQR (25–75%) values are reported. The last column lists the pairs of conditions with statistically significant differences, with * for $p < 0.05$ or ** for $p < 0.01$

Sit Down	%	T15	T20	T25	T30	$p < 0.05$ (*); $p < 0.01$ (**)
$\overline{M}_{h,max}$	50	0.0064	0.0070	0.0062	0.0053	
[*BWH*]	25	0.0038	0.0028	0.0020	0.0026	
	75	0.0086	0.0100	0.0111	0.0103	
$\overline{M}_{k,max}$	50	0.0231	0.0227	0.0213	0.0233	
[*BWH*]	25	0.0128	0.0143	0.0168	0.0150	
	75	0.0254	0.0245	0.0265	0.0258	
$\overline{F}_{v,max}$	50	0.536	0.482	0.507	0.510	
[*BW*	25	0.470	0.433	0.414	0.416	
	75	0.674	0.544	0.629	0.607	
$\overline{F}_{h,max}$	50	0.076	0.054	0.066	0.070	V15-V20*
[*BW*]	25	0.052	0.033	0.040	0.040	
	75	0.110	0.104	0.113	0.104	
$\overline{W}$	50	$-$0.474	$-$0.444	$-$.454	$-$0.465	
$[BW \Delta z_G]$	25	$-$0.543	$-$0.493	$-$0.580	$-$0.543	
	75	$-$0.392	$-$0.335	$-$0.320	$-$0.318	

All tables with computed metrics for all participants and conditions are available as Supplementary Material (Additional File 1 to Additional File 20).

## Appendix E: Participant comments on the trajectories

This section compiles spontaneous comments by the participants collected after each assistance condition trial in Block 03, where each item corresponds to the comments of a different participant:

- Static Handle Condition (*SH*):
  - helps less than the others
  - doesn’t make sense for sitting down; causes unusual stretch in the wrist
  - shoulder joints don’t feel comfortable
  - relative to other trajectories, needed more effort; works well for sitting down provided the rollator can support the movement
- C-Shape Trajectory (*IC*):
  - moves down too much causing back pain; feels uncomfortable and unnatural
  - better for sitting down; too much effort while standing up; handle goes too far forward
  - (the participant used a swearword when referring to this trajectory)
  - for sitting down, works only when lower limbs are completely relaxed; feels like being dragged out of the chair for standing up
  - felt unstable due to changing momentum
- L-Shape Trajectory (*IL*):
  - feels like loosing balance and unsupported when moving forward from sitting
  - the IC shape feels smoother than the IL shape, especially for sitting down
  - the movement felt robotic
  - going down (vertical) was better while sitting down. Horizontal was slow
  - sitting down felt unstable; pressure on the knee; Going down too fast while sitting
  - feels unnatural; worse for standing up; abrupt change in direction
  - standing up is same as IC shape; sitting down is weird as it ‘falls down’; better than IC shape
  - not intuitive; needs adaptation
  - for sitting down, works only when lower limbs are completely relaxed; feels like being dragged out of the chair for standing up, same as IC shape
  - used a lot of lower limb during standing up; needed to squat while sitting down which felt unnatural; lot more load on the arms
- Straight Trajectory (*ST*):
  - sitting down is a little slow
  - felt very stable; requires a lot of force while standing up; standing up isn’t linear
  - felt like falling on the chair while sitting down
  - felt forced pulling, rather than assisting
  - better than L shape, more intuitive
  - felt stable while sitting down but had to do something extra for stabilization; liked the fact that the starting and ending is slower
  - some effort required at the end while sitting down; too fast for elderly people; need to be adjusted for speed
- S-Shape Trajectory (*SS*):
  - standing up easier than sitting down (for all trajectories until now)
  - sitting down should be slower than standing up
  - more comfortable and natural overall; better for standing up
  - was helpful and comfortable; sitting down was best (among all trajectories)
  - gentle start helps to get ready for the activity
  - felt a little unstable during sitting down in the beginning because of the faster backward motion
